# A multi-scale probabilistic atlas of the human connectome

**DOI:** 10.1038/s41597-022-01624-8

**Published:** 2022-08-23

**Authors:** Yasser Alemán-Gómez, Alessandra Griffa, Jean-Christophe Houde, Elena Najdenovska, Stefano Magon, Meritxell Bach Cuadra, Maxime Descoteaux, Patric Hagmann

**Affiliations:** 1 Connectomics Lab, Department of Radiology, Centre Hospitalier Universitaire Vaudois (CHUV) and University of Lausanne (UNIL), Lausanne, Switzerland; 2 Center for Psychiatric Neuroscience, Department of Psychiatry, Centre Hospitalier Universitaire Vaudois (CHUV) and University of Lausanne (UNIL), Prilly, Switzerland; 3 Department of Radiology and Medical Informatics, University of Geneva, Geneva, Switzerland; 4 Medical Image Processing Laboratory, Neuro-X Institute, Ecole Polytechnique Fédérale de Lausanne (EPFL), Geneva, Switzerland; 5 Leenaards Memory Centre, Lausanne University Hospital and University of Lausanne, Lausanne, Switzerland; 6 Sherbrooke Connectivity Imaging Lab, Sherbrooke University, Sherbrooke, Canada; 7 Department of Radiology, Centre Hospitalier Universitaire Vaudois (CHUV) and University of Lausanne (UNIL), Lausanne, Switzerland; 8 Medical Image Analysis Laboratory (MIAL), Centre d’Imagerie BioMédicale (CIBM), Lausanne, Switzerland; 9 Signal Processing Laboratory (LTS5), Ecole Polytechnique Fédérale de Lausanne (EPFL), Lausanne, Switzerland; 10 Pharma Research and Early Development, Roche Innovation Center Basel, F. Hoffmann-La Roche Ltd., Basel, Switzerland

**Keywords:** Brain, Biomedical engineering

## Abstract

The human brain is a complex system that can be efficiently represented as a network of structural connectivity. Many imaging studies would benefit from such network information, which is not always available. In this work, we present a whole-brain multi-scale structural connectome atlas. This tool has been derived from a cohort of 66 healthy subjects imaged with optimal technology in the setting of the Human Connectome Project. From these data we created, using extensively validated diffusion-data processing, tractography and gray-matter parcellation tools, a multi-scale probabilistic atlas of the human connectome. In addition, we provide user-friendly and accessible code to match this atlas to individual brain imaging data to extract connection-specific quantitative information. This can be used to associate individual imaging findings, such as focal white-matter lesions or regional alterations, to specific connections and brain circuits. Accordingly, network-level consequences of regional changes can be analyzed even in absence of diffusion and tractography data. This method is expected to broaden the accessibility and lower the yield for connectome research.

## Abstract (summary)

**Table:** 

Measurement(s)	brain connectivity measurement
Technology Type(s)	Fiber tracking

## Background & Summary

The human brain is a complex system that can be efficiently represented as a network of gray matter (*GM*) regions interconnected by white-matter (*WM*) bundles^1–3^, named the human connectome. This network representation proved to be relevant in many fields of cognitive and clinical neuroscience^4–6^. Cognitive processes rely on the dynamic interaction between interconnected elements in neuronal networks, and so can cognitive and behavioral impairments as well as pathologic processes be explained by general or specific network failures^7,8^. Accordingly, following the current trend of network neuroscience, the knowledge and characterization of the connectome underlying brain processes is essential.

Since the first connectome formulations^1–3^, major progress has been achieved in the field of MRI data acquisition, post-processing, and validation^9–12^. It remains that proper, high quality connectomics is demanding in terms of equipment and expertise. Also, there is a large body of neuroimaging experiments and data that have been or will be collected that are not primarily dedicated to, or are technically not suited for, connectomics analysis, but that would potentially benefit from a network-oriented and/or connection-specific analysis *a posteriori*.

The advent of diffusion weighted imaging (*DWI*) and DWI-based tractography has opened new perspectives on the study of WM neuroanatomy, enabling the delineation of individual fiber tracts. Diffusion MRI data can be aggregated across multiple subjects to construct both DWI-based templates and WM parcellations. The development of DWI-based templates relies on suitable co-registration algorithms to match local diffusion orientation information across subjects. While different multi-step co-registration procedures have been proposed, including linear^13,14^, nonlinear^15,16^ and/or diffeomorphic^17–19^ transformations, this operation remains particularly challenging given the high dimensionality of diffusion information and could lead to loss of inter-subject variability at the voxel level^20,21^. Furthermore, diffusion tensor imaging (*DTI*) has been used for the development of several diffusion-based templates^22^, and few High Angular Resolution Diffusion Imaging- and Diffusion Spectrum Imaging-based templates^17–19,21,23,24^ have been proposed. With its Gaussian assumption, DTI limits accuracy in crossing fiber areas compared to more advanced DWI techniques.

Diffusion templates can be input to tractography algorithms to delineate WM trajectories and build WM parcellations^14,18,24^ or connectivity atlases^25^. Alternatively, tractography can be run at the subject level to create representative statistical maps of major tracts over a population, providing additional probabilistic information^26–29^. In general, specific tract delineation is achieved by filtering the whole-brain tractograms with selected regions of interest, even though voxel-to-voxel connectivity^30^ and clustering techniques^18^ have been proposed. These approaches often deliver well-known WM bundles (e.g., cortico-spinal tract, fornix, etc.^27,31^) or specific brain subnetworks selected on the basis of anatomical or functional information (e.g., cerebellar connectivity, sensorimotor network, etc.^21,26,32–34^) and may exclude valid connections that are poorly described in literature. Atlases based on specific tracts’ filtering enable “virtual dissections” of WM architecture^35^ but are not specifically tuned to connectomics research which relies on complete, whole-brain network information. Conversely, whole-brain connectivity atlases such as the Brainnetome^36^, the connectome IIT Human Brain Atlas^25^ or the population-averaged atlas^18^ provide structural connectivity information between all GM regions in the brain.

With the present work, we intend to deliver a multi-scale connectivity atlas named '*MultiConn*' that provides population-level whole-brain connectomics information and statistics and allows to perform customized connectomics analyses with any brain imaging data (without the need for diffusion MRI *per se*). To this end, we develop a probabilistic multi-scale atlas of the human brain connectome that is derived from a cohort of normal adults from the Human Connectome Project (*HCP*)^37^. The atlas is referenced in standard MNI (*Montreal Neurological Institute*) space with a high-resolution T1-weighted image. Accordingly, multimodal brain images can be aligned to the atlas, and individual connectivity matrices can be computed and put in relation with clinical or cognitive features. The atlas and its associated open-source code enables the interested user to easily perform several kinds of brain connectivity analysis even in absence of DWI and tractography data. These include (but are not limited to) atlas-based network analysis of quantitative T1-weighted or Magnetization Transfer Imaging volumes, with generation of multi-contrast scalar-weighted networks; assessment of WM lesion load-such as inflammatory lesions in multiple sclerosis patients or cerebrovascular WM changes in older adults-onto whole-brain connections or specific brain subnetworks (‘dysconnectome’ analyses); quantitative (scalar-based) analysis of bundles that intersect multiple regions of interest. Notably, the atlas allows to easily project alterations of the WM to specific brain connections and understand their impact onto specific cortical and subcortical structures or brain circuits.

Compared to previous approaches, the strengths of the connectome atlas proposed in this work rely on: (i) A focus on whole-brain WM connectivity for network-oriented analyses, in contrast to specific tracts or circuits known from literature^26,31,32^; (ii) A multi-scale assessment of brain connectivity: compared to other whole-brain atlases based on single-scale GM parcellations (e.g., Brainnetome^36^, IIT Human Brain Atlas^25^), the MultiConn allows to retrieve subject-specific connectivity matrices across four scales of investigation including 95 to 473 GM regions of interest, thus enabling connectivity analyses at different granularities referenced to a well-established anatomical parcellation (scale 1 corresponds to the Desikan-Killiany parcellation^38^ and integrates subcortical and thalamic structures^39^); (iii) A particular attention to connectivity estimation techniques: the MultiConn atlas implements extensively validated methods aimed at reducing biases such as crossing fiber artefacts (present in tensor-based atlases^40^) and gyral termination biases^41^. To this end, the MultiConn implements Constrained Spherical Deconvolution^42^ combined with anatomically constrained Particle Filtering Tractography seeded at the GM-WM interface and automatic streamline outliers’ rejection which, together, reduce shape, length, volume, and gyral termination biases of the reconstructed connections^43^. All methods used to construct the MultiConn atlas are shared as open-source code. (iv) Straightforward accessibility to single-connection probabilistic maps, ease of usage and flexibility: We share hundreds-of-thousands probabilistic WM bundles in a convenient and memory-efficient way through the Hierarchical Data Format (*HDF5*). Open-source, Python-based software is provided to apply the atlas within different research scopes, including multimodal connectome analysis, group- or individual-to-group comparison, and estimation of WM lesions’ impact on the connectome. We note that, besides the MultiConn, a few whole-brain connectivity atlases exist that allow to perform customized connectomics analyses^18,25,36^. These atlases differ in terms of GM parcellations, methodological approaches to atlas construction, and type and format of shared data and software. Future work is encouraged to compare whole-brain connectivity atlases-including the MultiConn-in terms of connectivity analyses’ outcome and applicability.

In this manuscript, we detail the technical development to construct the MultiConn atlas and describe several technical validations, including the application of the atlas to an external dataset. Our development is intended to support the broadest community of fundamental and clinical neuroscientists in performing high-end connectomics research.

## Methods

### Processing pipeline for the human connectome atlas

**Fig. 1:**
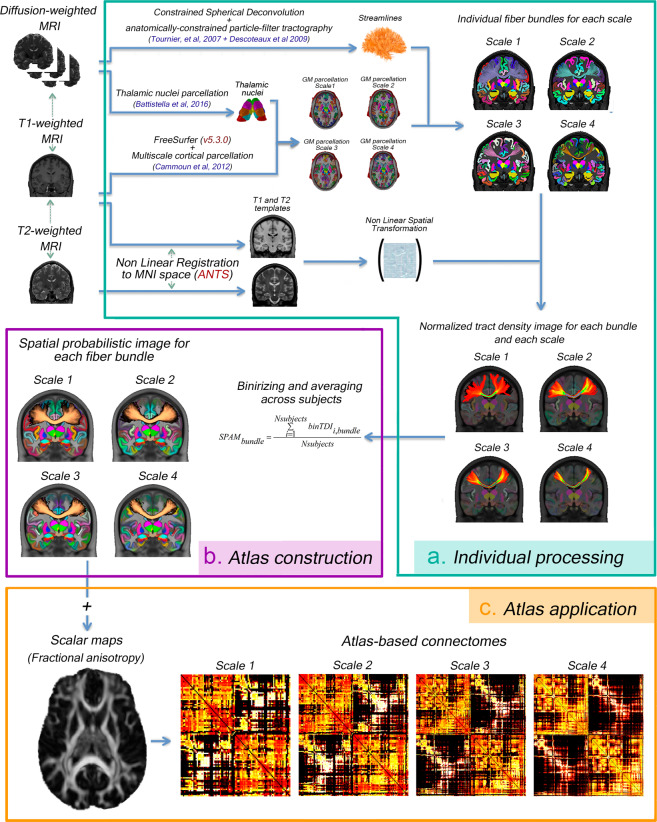
Processing workflow to create the multiscale probabilistic atlas of the white matter (*MultiConn*). (**a**) Processing steps applied to each subject. (**b**) Spatial probability map for a bundle in each of the scales. (**c**) Atlas-based connectomes computed using the developed multi-scale atlas.

The technical pipeline for the construction of the human connectome atlas is graphically summarized in Fig. 1. Briefly, each subject T1-weighted brain volume was segmented according to a four-scale GM parcellation^44,45^ (including a diffusion-based segmentation of thalamic nuclei^39^) for which the multi-scale atlas is available. Robust estimation of individual white matter bundles between pairs of GM regions was achieved from diffusion weighted imaging using constrained spherical deconvolution (*CSD*)^42,46^ and anatomically-constrained Particle Filtering Tractography (*PFT*)^43^ seeded from the GM-WM interface. The resulting PFT tractogram was carefully dissected in individual white matter bundles connecting pairs of gray matter regions.

T1-weighted (T1w) and T2-weighted (T2w) images were aligned to MNI space (ICBM 2009c Nonlinear Asymmetric 1 × 1 × 1 mm^47^) using a multimodal spatial registration framework^48^ and the resulting affine and nonlinear spatial transformations were applied to each scale-specific native WM bundle to map them to this stereotactic space. Finally, the individual bundles in MNI space were averaged across the subjects to build the scale-specific WM bundles’ spatial probabilistic anatomical maps. All these methodological steps are described in detail in the following sections and are available in public scripts.

#### MRI acquisition

##### Atlas dataset

From the one hundred unrelated subjects of the HCP dataset (*U100*), only the subjects belonging to the releases Q1, Q2 and Q3^12,37^ were selected, for a total of seventy subjects. From this cohort, four subjects were discarded because of different technical reasons. For three of them, the thalamic clustering failed to provide the expected segmentation pattern and for one the spatial alignment, obtained by the registration of the streamlines to MNI space, was not accurate. Thus T1w, T2w and DWI of a final cohort of 66 healthy subjects (age range 22 to 36 yo, 29 males) were used to build the publicly available, probabilistic multi-scale atlas of the human connectome. Each subject was scanned on a Siemens 3 T Skyra scanner in Washington University or University of Minnesota. T1w sagittal images were acquired using a Magnetization-Prepared Rapid Acquisition Gradient Echo (*MPRAGE*) sequence with 3D inversion recovery, echo time (*TE*) = 2.14 ms, repetition time (*TR*) = 2400 ms, inversion time (*IT*) = 1000 ms, flip angle (FA) = 8°, Bandwidth (*BW*) = 210 Hz per pixel, echo spacing (*ES*) = 7.6 ms, gradient strength = 42 mT/m, field of view (*FOV*) = 180 × 224 × 224 mm^3^, voxel size = 0.7 × 0.7 × 0.7 mm^3^ and acquisition time 7 min 40 s. T2w sagittal images were acquired using 3D T2 Sampling Perfection with Application-optimized Contrast by using flip angle Evolution (SPACE) sequence with TE  = 565 ms, TR = 3200 ms, BW = 744 Hz per pixel, ES = 3.53 ms, turbo factor = 314, FOV = 180 × 224 × 224 mm^3^, voxel size = 0.7 × 0.7 × 0.7  mm^3^ and acquisition time 8 min 24 s. Multi-slice echo planar imaging (*EPI*) with multi-band (*MB*) excitation and multiple receivers were acquired with TE = 289.5 ms, repetition time TR = 5520 ms, FA = 78°, refocusing flip angle (*rFA*) = 160°, BW = 1488 Hz per pixel, multiband factor = 3, ES = 0.78 ms, gradient strength = 100 mT/m, FOV = 210 × 180 × 138  mm^3^, voxel size = 1.25 × 1.25 × 1.25 mm^3^ and b-values = 0, 1000, 2000 and 3000 s/mm^2^. Each gradient table includes approximately 90 diffusion–weighted directions plus 6 b0 acquisitions interspersed in each run. The acquisition time was around 63 min. More details about the MRI acquisition protocols are described in Van Essen *et al*.^37^.

#### Image processing steps for building the WM atlas

##### Gray matter parcellation

Alongside the native T1w and T2w images, the HCP database also provides the FreeSurfer (v5.3.0) outputs computed by an optimized processing pipeline^49^. These outputs contain the cortical surfaces (pial and white), the cortical maps (thickness and curvature), and a parcellation of the cortical surfaces containing 34 regions for each hemisphere based on the atlas developed by Desikan *et al*.^38^. This cortical parcellation corresponds to the first scale of the multiscale cortical parcellation methodology developed by Cammoun and colleagues^44^.

According to the latter method, each region of the right hemisphere of the Desikan cortical parcellation (*scale 1*) was subdivided in sub-regions with uniform surface area of 1.5cm^2^ approximately, representing the finest parcellation (*scale 5*) of the pial surface in stereotactic space (FreeSurfer *fsaverage* space). Then, scales 4, 3 and 2 were obtained by a successive grouping of neighboring regions at the next higher resolution scale. Scale 5 was discarded for the atlas construction because of higher spatial-location variability of individual fiber bundles in MNI space compared to the other four scales. At scale 5, parcels are small and robust connectivity estimation is challenging^50^. The cortical parcellations of the right hemisphere for the remaining scales (2, 3 and 4) were then mapped onto the left pial surface in order to obtain a symmetric organization of the cortical regions. The boundaries of the projected parcellation for scale 1 were aligned to the boundaries of the original left cortical parcellation obtained by Desikan *et al*.^38^. Finally, the resulting transformation was applied to the projected parcellations of scales 2, 3 and 4 to create the symmetric multi-scale cortical parcellation. At the end of the process, four parcellations comprising 68, 114, 216 and 446 cortical regions, respectively, were mapped to each subject-specific space to obtain both the individual multi-scale surface-based cortical segmentation and the corresponding volumetric parcellation of the cortex (see Fig. 1a). In order to get tractography termination masks suitable for the connectomes’ construction, the cortical gray matter regions were dilated toward the WM with a radius of 2 mm.

The volumetric parcellations were complemented with bilateral segmentations of different subcortical structures and the brainstem. The subcortical structures, including the striatal structures (caudate nucleus, putamen and nucleus accumbens), globus pallidum, amygdala, thalamus and hippocampus, and brainstem were obtained from FreeSurfer^45^. Additionally, each individual thalamus was subdivided into seven different nuclei using the framework proposed by Battistella *et al*.^39^. This approach employs the orientation distribution functions (*ODFs*) computed from the DWIs to subdivide each thalamus into seven thalamic nuclei: *ventral anterior*, *mediodorsal*, *lateral posterior-ventral posterior group*, *pulvinar medial-centrolateral group*, *ventrolateral*, *ventral posterior-ventrolateral group*. Eventually, the resulting multi-scale parcellation contained 95, 141, 243 and 473 gray matter regions which were used as seed regions in the tractography process.

The individual T1w images were also segmented using FAST to obtain a partial volume estimation (*PVE*) of the three tissue classes GM, WM and cerebrospinal fluid (*CSF)*^51^.

##### Tractogram reconstruction

The DWIs provided by the HCP were already preprocessed by a pipeline including the following correction steps^49^: intensity normalization, head motion correction (with gradient table rotation), eddy current and distortion corrections.

The native multi-scale GM parcellation and the tissue segmentation images were interpolated to the subject-specific diffusion space using nearest neighbor as interpolation method.

The corrected DWI of each subject was employed to fit a second order tensor for each voxel and compute voxel-wise scalar maps (*FA:* Fractional anisotropy and *MD*: Mean diffusivity) using Dipy^52^. The diffusion tensor was estimated with a weighted least-squares fitting using only the data corresponding to the lowest b-value shell (i.e., b = 1000 s/mm²)^53^.

The corrected DWIs were also used to estimate the intravoxel fiber orientation distribution function (*fODF*) by using the Constrained Spherical Deconvolution (*CSD*)^42^ approach implemented in Dipy. A single fiber response function was fixed for all subjects to [15, 4, 4] × 10^−4^ s/mm^2^, as recommended in^54,55^; a maximal spherical harmonics order of 8 and all b-value DWI data were used. The fODFs were input to the anatomically-constrained Particle Filtering Tractography algorithm^43^ to obtain the individual tractograms where 30 tractography seeds per voxel of the GM-WM interface from the PVE maps were selected. The script to perform the fiber tracking (*hcp_script_connectome_atlas_scilpy.sh*) is included in the online repository (https://github.com/connectomicslab/probconnatlas).

The PFT approach reduces length, shape and volume biases of reconstructed connections, it is robust to partial volume effects between GM, WM and CSF, and it ensures that streamlines stop at cortical or subcortical GM regions^43^. Only streamlines of length between 20 and 200 mm were kept, which resulted in whole-brain tractograms of approximately 2.5 M compressed streamlines (~500 Mb), depending on the brain size and number of voxels belonging to the GM-WM interface. The streamlines’ compression was done according to the compression pipeline proposed by Presseau and colleagues^56^ and implemented in scilpy (https://github.com/scilus/scilpy) with a maximum error of 0.2 mm^57,58^.

##### Connectivity estimation and white matter bundles extraction

An in-house connectivity tool was developed to carefully dissect the full PFT tractogram in all combinations of WM bundles based on the dilated multiscale parcellations. Firstly, the PFT tractogram of each subject was filtered (see Supplementary material, Supp 1) to extract the WM bundle *C*_*k,i,j*_ between each pair of GM regions *(i, j)* at parcellation scale *k* (see Fig. 1a). This step requires a careful definition of streamline cutting and termination rules and outlier rejection to provide anatomically meaningful WM bundles. Secondly, the streamlines from the WM bundle *C*_*k,i,j*_ considered as outliers (streamlines taking anatomically implausible paths) were automatically removed from the final bundle *C*_*k,i,j*_ using an algorithm that identifies streamlines creating loops (i.e., winding more than 360 degrees). Outliers are then detected using a hierarchical clustering approach based on QuickBundles^57,59^ with a tree-length threshold of 0.2^60^.

For each scale, the filtered WM bundles between each pair of GM regions were individually saved and used to build the WM atlas. Besides, individual connectivity matrices were also computed, where weights represent the number of streamlines (*NOS*) belonging to the bundle connecting each pair of regions.

##### Atlas construction

Individual T1w and T2w images were nonlinearly warped to their respective reference templates in MNI space using the multimodal registration approach implemented in Advanced Normalization Tools (*ANTs*)^61^. The T1w and T2w ICBM 152 brain templates (ICBM 2009c, 1 mm isotropic voxel size, nonlinear, asymmetric^47^) were used as reference images. The spatial geometric transformations were applied to map individual WM bundles to MNI space using the ANTs *antsApplyTransformsToPoints* subroutine (see Fig. 1a). High-resolution volumetric tract density images (*TDI*)^62^ containing the number of streamlines passing through each voxel were computed for each scale, bundle, and subject. By averaging individual TDIs across subjects, two different images were obtained for each WM bundle at each scale: (1) the mean TDI image, and (2) the spatial probabilistic anatomical map (*SPAM*) of the bundle. Each bundle’s SPAM was computed by binarizing the individual TDIs (lower threshold equal to one streamline), summing the resulting individual bundle masks across the subjects, and dividing the value of each voxel by the number of subjects (66 in this case). These maps (one for each brain connection, at different scales) are voxel-wise inter-subject consistency maps which represent the probability that a given voxel is traversed by at least one streamline of the considered connection (Fig. 1b). A **voxel-wise probability threshold** can be set by the user of the atlas to exclude low-probability voxels.

The final list of bundles included in the atlas for each scale was defined by setting an **inter-subject consistency threshold** representing how many subjects have a non-zero fiber count between a given pair of brain regions. The thresholding was implemented using the methodology proposed by Betzel *et al*.^63^, which creates a group-representative network by discarding the connections that are not present in a minimum percentage of subjects (consistency threshold) while separately preserving the connectivity density and geodesic length of intra- and inter-hemispheric connections. To this end, a length matrix was computed, where each entry is the mean geodesic length of the streamlines connecting a regions’ pair. This thresholding approach guarantees that the resulting atlas-based connectivity matrices have connectivity density similar to the ones of individual subjects, while correcting for possible length-biases linked to the tractography algorithms^64^. The final numbers of bundles included in the atlas are 4222, 9232, 26502 and 89840 for scales 1, 2, 3 and 4 (Figure S1), which correspond to whole-brain connectivity densities of 94.5, 93.5, 90.3 and 80.5%, respectively.

**Fig. 2:**
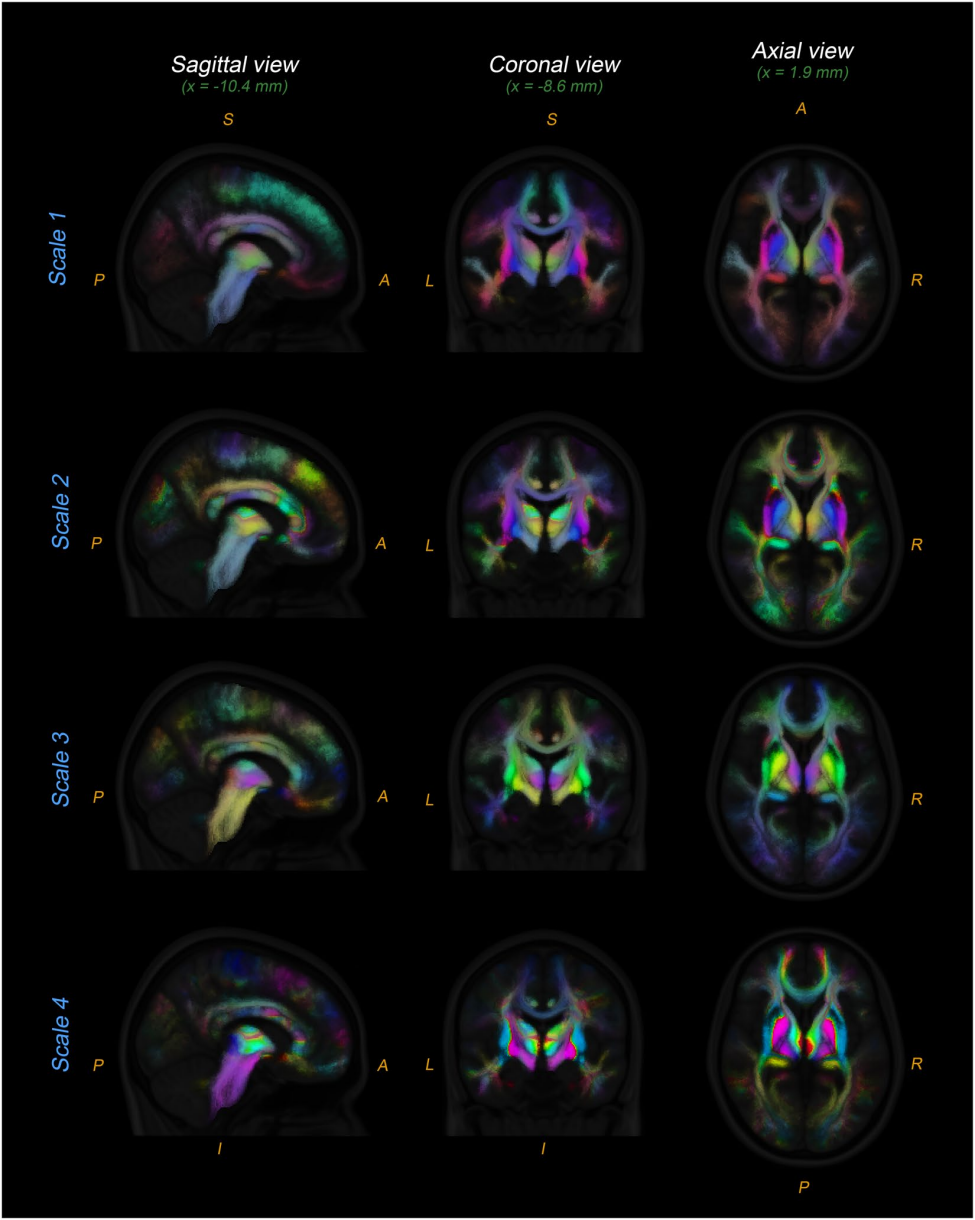
Orthogonal views of the probabilistic multi-scale white matter bundles atlas. Only a subset of the bundles intercepting the selected planes are displayed. Different colors indicate different WM bundles and the intensity of the color represents the probability of the voxel to belong to that bundle. Note that, when a voxel contains multiple WM bundles, the colors are mixed, i.e., the final color of the voxel is a weighted average of the colors of the fiber bundles passing through it.

1Different views of the resulting probabilistic white matter atlas for the four scales are presented in Fig. 2. The bundles are represented in different colors, and the intensity of the color in single voxels is proportional to the voxel probability (across the subjects) of belonging to each bundle. Regions with blurred or mixed colors are regions containing the intersection or spatial confluence of multiple fiber bundles. This effect can be clearly observed in the corpus callosum (Fig. 2, sagittal views). We note that, due to the high anatomical variability among subjects, the spatial reliability of the registration algorithm is lower in juxtacortical regions compared to deep white matter regions. Registration inaccuracies can increase spatial uncertainty and decrease the inter-subject voxel probability of belonging to each bundle.

Finally, a set of Python-based functions complements the atlas and facilitates the computation of connection-specific scalar values from different image contrasts (e.g., DWI-scalar, quantitative T1, quantitative T2, etc.), allowing (cross-modal) connectomics analyses in the absence of tractography data. The function that applies the atlas to custom datasets allows the user to tune the voxel-wise probability threshold and the inter-subject consistency threshold, which represent the only two free-parameters of the atlas (see Technical Validation and Supplementary material, Supp 5 for suggested parameters’ choice).

## Data Records

A summary of the data records related to this study is given in Table 1.

**Table 1:** Demographic information of the subjects and summary of the data records related to this work.

Dataset	Human Connectome Project (*100 unrelated subjects*)	Human Connectome Project (*Test-Retest dataset*)
***Number of subjects***	66	44
***Gender (F/M)***	38/28	31/13
***Age range (years):***		
• *22*–25	7	4
• 26–30	25	13
• 31–36	33	27
• *>36*	1	
***Release:***		
• *Q1*	15	16
• *Q2*	21	6
• *Q3*	30	2
• *MEG2*		5
• *S500*		10
• *S900*		4
• *S1200*		1
***Used modalities***	T1w, T2w and dMRI	T1w and dMRI
***Study type:**** Time between acquisitions (mean, [min, max]) days*	Cross-sectional	Longitudinal (2 acquisitions) 135.15 [18, 328]
	—	
***Experimental usage***	Building the atlas	Technical validation of the atlas
***Provided output***	Probabilistic multi-scale WM bundle atlas and a scale-specific color-coded 4D image of the WM bundles atlas	—
*Provenance*	refer to https://db.humanconnectome.org	
*Available from*	https://db.humanconnectome.org	

### Data records as a contribution

The main contribution of the presented work is a single data record containing different files with different file formats.

#### HDF5 files

The developed multi-scale atlas is presented in four Hierarchical Data Format (HDF5) files (**.h5** extension), each one containing the probabilistic white matter bundles for one of the four connectivity scales. As summarized in Table 2, each HDF5 file contains three different groups of datasets: (1) **header**, (2) **matrices** and (3) **atlas**.

**Table 2:** Content and internal organization of the HDF5 atlas files.

HDF5 Group	HDF5 Dataset	Description
header (8 datasets)	nsubjects	number of subjects used to build the atlal (nsubjects = 66)
	dim	image dimensions
	voxsize	voxel size (mm)
	affine	position of the image array data in MNI space
	gmregions	anatomical labels of GM regions
	gmcodes	numerical IDs of GM regions
	gmcolors	RGB colors of GM regions
	gmcoords	spatial coordinates in MNI space of GM region centroids
matrices (3 datasets)	consistency	N_GM_ × N_GM_ matrix reporting the number of subjects having at least one streamline for each specific brain connection
	numbStlines	N_GM_ × N_GM_ matrix reporting the average number of streamlines across subjects for each specific brain connection
	length	N_GM_ × N_GM_ matrix reporting the average streamlines’ length across subjects for each specific brain connection
atlas (N_bundle_ datasets)	a_b	N_a_b_ × 4 matrix, with N_a_b_ number of voxels in the specific bundle connecting GM regions a and b. The first three column are the voxel X,Y,Z coordinate in MNI space; the fourth column is the subject consistency (bundle’s probabilistic information)

There is one HDF5 file per connectivity scale. RGB = red, green, blue; GM = gray matter; N_GM_ = number of gray matter regions (95, 141, 243 and 473 for scales 1 to 4, respectively); N_bundle_ = number of bundles (brian connections) reconstructed for a given atlas scale (4222, 9232, 26502 and 89840 for scales 1 to 4, respectively).

The **header** group contains the number of subjects employed to build the atlas and the required information to pass from the HDF5 format to Nifti-1 file format. This data is organized in different datasets: ***subjects***: number of subjects; ***dim***: image dimensions; ***voxsize***: voxel dimensions; ***affine***: position of the image array data in MNI space. The header group also contains scale-specific information about the gray matter regions employed to separate the bundles (***gmregions***, ***gmcodes***, ***gmcolors*** and ***gmcoords***: names, codes, RGB (red, green and blue) colors triplets and spatial coordinates in MNI space, respectively). This information is useful for visualization purposes and key to establish the relationship between the WM bundles and the real brain anatomy.

The **matrices** group contains three relevant connectivity matrices computed from the subject’s sample used to create the multi-scale atlas: (1) ***consistency***, (2) ***numbStlines*** and (3) ***length matrices***. Each element of these matrices represents a connection between a pair of GM regions and contains: (1) the number of subjects for which at least one streamline was found to connect the two GM regions (consistency matrix); (2) the average number of connecting streamlines across subjects (numbStlines matrix); (3) the average geodesic length of the connecting streamlines across subjects (length matrix). The consistency and numbStlines matrices for each scale are displayed in the supplementary material (Figure S2).

Finally, the ***atlas*** group is composed of datasets, one for each WM bundle, which contain the coordinates and subject consistency (probabilistic information) of the voxels belonging to the bundle. Specifically, each dataset contains a Nx4 matrix where N is the number of voxels belonging to the bundle. The first three columns are the X, Y, Z voxel coordinates in MNI space. The fourth column is the ‘subject consistency’, i.e., the number of subjects for which at least one streamline passes through the specific voxel. The names of these datasets are defined according to the codes of the GM regions connected by the bundle (e.g., 1_10: bundle connecting regions 1 and 10).

#### Nifti-1 files

In addition to the four HDF5 files, the following complementary images are provided: (1) a 3D Nifti-1 image containing the average number of streamlines passing through each voxel across subjects; (2) four 3D Nifti-1 images (one per scale) with the number of bundles passing through each voxel across subjects; (3) four color-coded Nifti-1 images (one per scale) with colors uniquely representing different white matter bundles. The latters are 4D volumetric Nifti-1 images where the fourth dimension represents the red, green and blue channels, respectively. For visualization purposes, the colors of the intra-hemispheric bundles are symmetric between hemispheres (see Fig. 2). These files, together with the MNI T1w template, can be opened with any available Nifti visualization tool such as *FSLeyes, AFNI, or Micron*.

The data record derived from this work is available through Zenodo^65^.

### Original datasets used

The used HCP data is provided by the Human Connectome Project, WU-Minn Consortium (Principal Investigators: David Van Essen and Kamil Ugurbil; 1U54MH091657) funded by the 16 NIH Institutes and Centers that support the NIH Blueprint for Neuroscience Research; and by the McDonnell Center for Systems Neuroscience at Washington University. All the participants provided written informed consents. All participants provided written informed consents.

## Technical Validation

### Evaluation dataset

To assess the validity of the connectome atlas with respect to its application to user-specific imaging data, we characterized the differences appearing when segmenting the WM bundles of an independent dataset with the developed atlas compared to a tracking-based parcellation. To this end, an independent cohort of 44 healthy subjects from the *HCP Test-Retest* dataset (44 subjects with baseline and follow-up acquisitions) with T1w, T2w and DWI data was selected. These subjects were scanned with the same acquisition protocol as the subjects employed to build the probabilistic WM atlas but none of them was used to construct the atlas.

The original and the evaluation datasets are freely available at https://db.humanconnectome.org. The IDs of all the subjects, their age and gender are provided in two comma separated files (**.csv**) stored in the same Zenodo repository as the developed atlas^65^.

### Connectivity estimation

For each subject, and both baseline and follow-up MRI acquisitions, the individual WM bundles were parcellated using both the connectome atlas and a tracking-based segmentation. The tracking-based segmentation was performed by using two different fiber tracking approaches: (1) *SD_STREAM* (Streamlines by using Spherical Deconvolution)^66^ and (2) *iFOD2* (Second-order Integration over Fiber Orientation Distributions). These two approaches were selected to evaluate the impact of using deterministic (SD_STREAM) or probabilistic (iFOD2) tractography on the comparison with the atlas-based segmentation (see Supplementary material, Supp 2).

Both tracking-based segmentation approaches were performed using the Connectome Mapper 3 (*CMP3*) image processing suite (https://connectome-mapper-3.readthedocs.io/en/latest/). The methods described in previous sections were employed to obtain the multiscale GM parcellations, the intravoxel fODFs and the diffusion tensors with their corresponding scalar maps. The resulting fODFs were input to both fiber tracking algorithms to obtain the streamlines distribution. Finally, the multi-scale structural connectivity matrices were computed. The connection strength between each pair of GM regions was quantified as the number of streamlines connecting the regions. In addition, for each scalar map (FA map in this case), the connection strength was also quantified as the mean scalar value along the bundle connecting each pair of GM regions (Supplementary material, Supp 1).

The atlas-based segmentation was obtained by segmenting each T1w image in three different tissue classes (GM, WM and CSF) and non-linearly registering the T1w to MNI space using ANTs. The resulting spatial transformation was then applied to map the individual FA images to MNI space. Finally, four structural connectivity matrices (one *per* scale) weighted by the mean FA along each bundle were obtained using the developed multi-scale connectome atlas. These operations can be performed using the set of Python tools shared with the atlas (https://github.com/connectomicslab/probconnatlas). The individual atlas-based FA matrices for all the scales were created using an inter-subject consistency threshold equal to 30% and a voxel-wise probability threshold of 0.3. This parameters’ combination results in the highest correlation between the atlas-based and the tracking-based FA matrices and is therefore suggested for user-specific atlas usages (Supplementary material, Supp 5).

### Evaluation: cortical coverage of the developed atlas

The individual WM volumes of the 44 HCP subjects were parcellated using both the developed atlas and the tracking-based methods. Then, different metrics and tests were proposed to assess, qualitatively and quantitatively, the accuracy and reproducibility of the connectivity matrices obtained using the atlas-based approach in comparison to the tracking-based approaches.

The number of bundles reaching each vertex of the white-gray matter interface (white surface) was calculated to evaluate the cortical coverage of the white matter connections. The coverage was also assessed for gyral and sulcal regions separately to detect possible biases in the tracking method to reach both cortical regions. To this end, the individual cortical white surfaces of the 44 test-retest subjects were parcellated into gyri and sulci by thresholding the curvature values provided by FreeSurfer. The curvature threshold was set to 0, which corresponds to the surface point-of-inflexion between gyri and sulci. This is the point where the cortical surface shifts from convex to concave or vice versa.

**Fig. 3:**
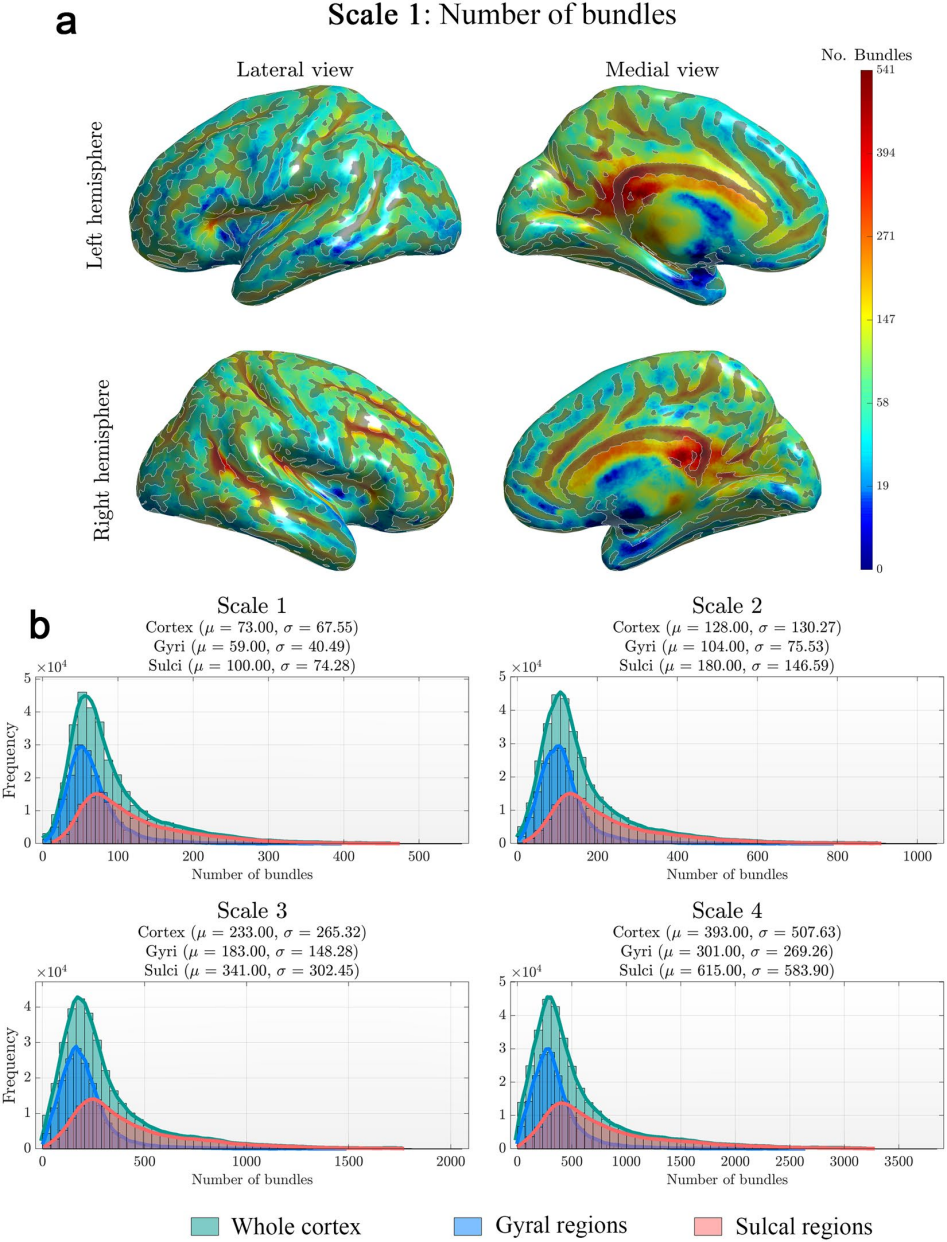
Cortical coverage of the scale 1 of the atlas projected onto a single subject surface. The cortical coverage is represented by the total number of bundles reaching each point of the gray-white matter interface. (**a**) The results are displayed over the inflated surface using a logarithmic scale to enhance the visualization in sulcal regions and vertices with low number of bundles. The cortical regions belonging to sulcal areas are outlined in the figure. (**b**) Histogram of the number bundles along the cortex for each scale. The histograms are presented for three different regions of interest: the whole cortical surface, the gyral regions and the sulcal basins. **Note:** The cortical coverage for the rest of the scales are shown in the supplementary material (Figures S3, S4 and S5).

The cortical coverage of the developed atlas can be appreciated in Fig. 3 (but see also Supplementary material, Supp 4). The cortical projection of all the bundles onto the individual cortical surface of a subject of the test-retest dataset is depicted in Fig. 3a. The number of bundles reaching the sulcal regions is higher than the ones reaching the gyral regions. This effect is presented in Fig. 3b for all the scales. In some cases, the number of bundles reaching the sulcal basins is the double of the ones reaching the gyral regions (scale 4, gyri: µ = 301, sulci: µ = 615). This difference can be mainly explained by two factors: (1) the gyral termination bias inherent to tractography algorithms (although the PFT minimizes this bias)^67^, and (2) the decreased reliability of registration algorithms in juxtacortical regions^68^. Despite these factors, a dense spatial coverage of all cortical regions in all the scales is achieved, supporting the validity of the connectome atlas and its usage to compute accurate and meaningful scalar-weighted connectivity matrices. Each cortical area is reached by at least one atlas bundle at the resolution of the cortical-surface sampling, i.e., each cortical vertex is reached by a meaningful number of streamlines (Figures S6 and S7).

### Evaluation: reproducibility

The percentage of change in the connection-specific FA values between the test and retest acquisitions, segmented with both the connectome atlas or the tracking-based approaches, was obtained. This metric was calculated for each connection *(i, j)* and each scale *k* of the individual connectivity matrices (subject *s*) according to the following expression:

$$perC{h}_{s}\left(k,i,j\right)=\frac{F{A}_{s}^{2nd\;Acq}\left(k,i,j\right)-F{A}_{s}^{1st\;Acq}\left(k,i,j\right)}{F{A}_{s}^{1st\;Acq}\left(k,i,j\right)}\times 100$$

**Fig. 4:**
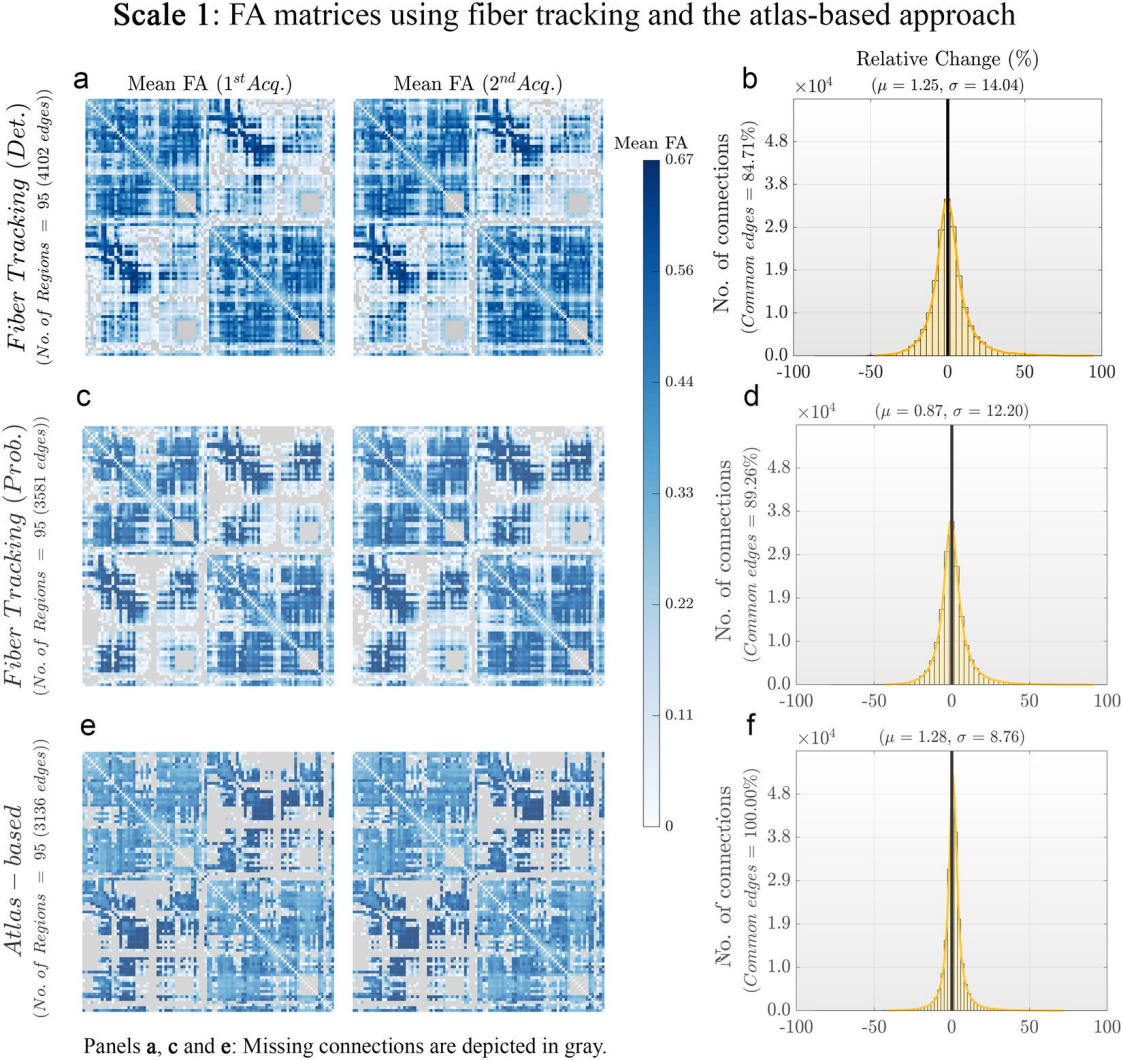
Mean FA matrices for both acquisitions and the percentage of difference between them obtained using tracking-based and atlas-based approaches for scale 1. (**a**) and (**c**) Mean connectivity matrices computed using two different fiber tracking approaches: 1) deterministic (SD_Stream) and 2) probabilistic (iFOD2). (**e**) Mean connectivity matrices obtained using the atlas-based approach. These matrices were computed for both acquisition and the connection strength between each pair of regions is given by the mean FA value along the bundle connecting them. (**b**), (**d**) and (**f**) Histograms of the percentage of difference between the connectivity matrices computed for both acquisitions. **Notes:** Individual tractography-based matrices were masked by the binary structure of the common edges, i.e., the connections present in both test and retest data (84.71% and 89.26% connections, respectively). Similar graphs for the rest of the scales can be consulted in the supplementary material (Figures S9, S10 and S11).

The mean connectivity matrices for scale 1 for both acquisitions, obtained using the atlas-based and the tracking-based approaches, are presented at Fig. 4a,c,e. Gray regions represent missing connections. Histograms of percentage change are displayed in Fig. 4b,d,f. Note that for tracking-based results (Fig. 4b,d), only common edges (i.e., edges present in both test and retest data) were used to generate the histograms of percentage change.

In general, the atlas-based connectivity matrices show spatial patterns similar to the ones obtained using the tracking-based approaches. The differences observed between the test and retest acquisitions were similar for the tracking-based and the atlas-based approaches (relative change <1.5%). The atlas-based matrices presented lower variability in the test-retest percentage change (percentage change standard deviation across connections σ = 8.76), compared to deterministic (σ = 14.04) and probabilistic (σ = 12.20) tracking-based approaches.

On one hand, tractography algorithms are known to be sensitive to experimental parameters, partial volume effects, noise, head size, and crossing fibers, and they are likely to produce a significant number of false connections because of streamline propagation errors^69^. For this reason, any quantitative metric based on fiber-tracking inherits these sources of variability which indirectly impact the test-retest reliability of results. Although we applied a tractography filtering approach to mitigate tractography biases (Supplementary material, Supp 1), the integration of latest developments such as Convex Optimization Modeling for Microstructure Informed Tractography (COMMIT2^70^)) among others^71^, could further improve connectome mapping. On the other hand, the atlas-based results are highly reproducible because the set of fiber bundles is selected a priori and assessed in the same way in both acquisitions. In this case, the registration algorithm and the voxel-wise probability threshold are the main sources of variability in the obtained connectivity matrices. Poor registration results in inaccurate placement of WM bundles and, therefore, in the selection of noisy voxels for the computation of mean FA values. Moreover, different voxel-wise probability thresholds lead to different spatial distributions of the same WM bundle, and thus to different sets of voxels for the computation of mean FA values.

### Evaluation: Differences between tracking-based and atlas-based FA matrices

Bland-Altman plots were created for each scale *k* and both test and retest acquisitions to evaluate the agreement between atlas-based and tracking-based approaches to generate FA-weighted structural connectivity matrices. Concretely, two types of plots were created: (1) the mean FA along the atlas bundles were compared to the mean FA along the bundles obtained using either deterministic or probabilistic fiber tracking, to determine their similarity and the validity of the atlas-based approach. These analyses were independently performed for both test and retest data. (2) The test-retest FA values obtained by using the atlas-based and the tracking-based approaches were compared to assess the methods’ test-retest reproducibility.

**Fig. 5:**
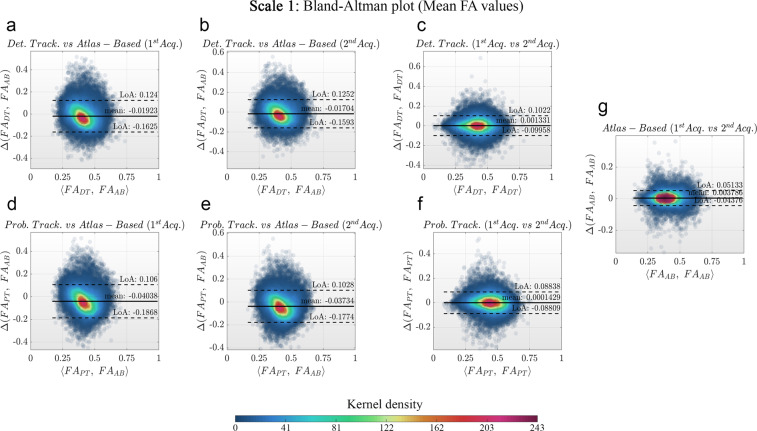
Bland-Altman plots displaying the bundles-wise FA differences between tracking-based and atlas-based approaches for the first scale of the developed multi-scale atlas. **(a)** and **(b)** Differences between deterministic tracking and atlas-based for both acquisitions of the test-retest dataset. **(c)** Differences in FA values between both acquisitions when using deterministic fiber tracking. **(d)** and **(e)** Differences between probabilistic tracking and atlas-based for both acquisitions of the test-retest dataset. **(f)** Difference in FA values between both acquisitions when using probabilistic fiber tracking. **(g)** FA differences between both acquisitions when using the atlas-based approach. **Notes:** LoA stands for level of agreements. The Bland-Altman plots for the scales 2, 3 and 4 are presented in the supplementary material (Figures S12, S13 and S14). Colors represent the probability density of the sample estimated using the closest 900 points.

The results of these analyses depicted negligible differences between methodologies (see Fig. 5). In all cases, the atlas-based mean FA values were lower than the one obtained using fiber tracking. This is related to the decreased reliability of registration algorithms in juxtacortical regions due to inter-subject anatomical variability. Locally poor registration causes juxtacortical voxels (with lower FA values compared to deep WM) to be taken into account for the computation of the mean FA along the bundle, thus decreasing the mean FA of the bundle itself.

For all scales, the level of agreement of FA values was higher for the inter-methods comparisons (tracking-based vs atlas-based) than for the intra-method comparisons (test vs retest). The agreement obtained for scale 4 was lower than the one obtained for the other scales.

### Evaluation: Correlation between tracking-based and atlas-based FA matrices

To complement the technical validation, various correlation analyses were performed between the connection-wise FA values obtained with the atlas-based and the tracking-based approaches. In all these analyses, the values from all the individual matrices were vectorized and concatenated, and two different correlation coefficients were computed.

**Fig. 6:**
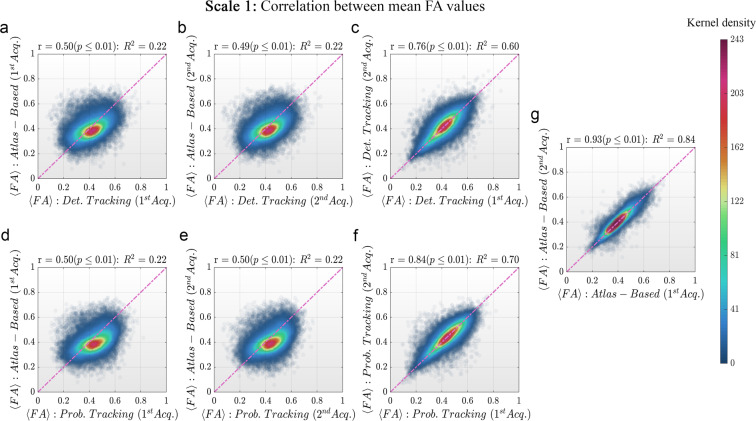
Bundles-wise FA correlations between tracking-based and atlas-based approaches for scale 1 of the developed multi-scale connectome atlas. **(a)** and **(b)** Correlations between deterministic tracking and atlas-based FA values for both acquisitions. **(c)** Correlation of FA values between both acquisitions using deterministic fiber tracking. **(d)** and **(e)** Correlations between probabilistic tracking and atlas-based for both acquisitions. **(f)** Correlation in FA values between both acquisitions using probabilistic fiber tracking. **(g)** FA correlation between both acquisitions using the atlas-based approach. **Note:** Dashed line represents correlation equal to one. Colors represent the probability density of the sample estimated using the closest 900 points. The correlation results for the rest of the scales are presented in the supplementary material (Figures S15, S16 and S17).

Firstly, Pearson’s correlation coefficients were computed to quantify the agreement between both inter-methods (tracking-based vs atlas-based) and intra-method (test vs retest) FA matrices (see Fig. 6). The p-values resulting from these correlations were corrected using false discovery rate (FDR^72^) and a q-value equal to 0.05. Secondly, the Lin’s Concordance Correlation Coefficients (CCC^73^) were used to assess the reliability between methods (see Table 3).

Results for scale 1 are depicted in Fig. 6 and Table 3 (results for the other scales are presented in the Supplementary material, Figures S15, S16 and S17).

**Table 3:** Lin’s concordance correlation coefficient for each comparison between FA matrices using both tracking-based and atlas-based approaches.

	*Lin’s Concordance Correlation Coefficients (CCC)*			
	Scale 1	Scale 2	Scale 3	Scale 4
	*CCC [CI]*	*CCC [CI]*	*CCC [CI]*	*CCC [CI]*
*Det. Tracking (1*^*st*^ *Acq.) vs Atlas-based (1*^*st*^ *Acq.)*	0.444 [0.439, 0.449]	0.441 [0.438, 0.445]	0.446 [0.444, 0.449]	0.381 [0.380, 0.383]
*Det. Tracking (2*^*nd*^ *Acq.) vs Atlas-based (2*^*nd*^ *Acq.)*	0.447 [0.442, 0.452]	0.443 [0.439, 0.447]	0.431 [0.428, 0.433]	0.369 [0.367, 0.371]
*Det. Tracking (1*^*st*^ *Acq.) vs Det. Tracking (2*^*nd*^ *Acq.)*	0.773 [0.770, 0.775]	0.769 [0.767, 0.771]	0.763 [0.762, 0.765]	0.772 [0.771, 0.773]
*Prob. Tracking (1*^*st*^ *Acq.) vs Atlas-based (1*^*st*^ *Acq.)*	0.390 [0.385, 0.395]	0.399 [0.395, 0.402]	0.434 [0.431, 0.436]	0.409 [0.407, 0.411]
*Prob. Tracking (2*^*nd*^ *Acq.) vs Atlas-based (2*^*nd*^ *Acq.)*	0.408 [0.403, 0.413]	0.419 [0.415, 0.422]	0.427 [0.424, 0.429]	0.402 [0.401, 0.403]
*Prob. Tracking (1*^*st*^ *Acq.) vs Prob. Tracking (2*^*nd*^ *Acq.)*	0.837 [0.835, 0.839]	0.831 [0.829, 0.833]	0.824 [0.823, 0.825]	0.815 [0.814, 0.816]
*Atlas-based (1*^*st*^ *Acq.) vs Atlas-based (2*^*nd*^ *Acq.)*	0.915 [0.913, 0.916]	0.916 [0.914, 0.917]	0.911 [0.910, 0.912]	0.963 [0.962, 0.964]

In all the analyses, a high correlation was observed for all the scales after correcting for multiple comparisons. The inter-methods correlation coefficients, for both acquisitions, were lower compared to the intra-method (test-retest) correlation coefficients. When increasing the number of bundles, i.e., the atlas scale, the correlation values tend to decrease. Note that high Pearson’s correlation values do not automatically imply a good agreement between the compared methods because they evaluate only the linear association of two sets of observations.

### Conclusion

In summary, reliable and reproducible connectivity matrices can be computed from custom data using the developed multi-scale probabilistic atlas of the human connectome. The resulting atlas-based connectivity matrices showed similar spatial patterns with highly correlated connection-wise FA values in a test-retest setting, close to the FA values observed in the tracking-based connectivity matrices.

The MultiConn atlas provides the neuroscientist with a grounded methodology to compute whole-brain multi-modal connectivity maps at multiple spatial granularities when diffusion weighted MRI data are absent, or when tractography is not possible or highly challenging (e.g., in presence of white matter lesions). Even though the connectome-atlas bundle maps can be prone to false positive artifacts, a complete probabilistic information of human connectivity is provided to the user at the best of current processing and tractography techniques, allowing for further data selection and filtering according to specific needs and research questions.

## Usage Notes

Firstly, the T1w images need to be non-linearly registered to MNI space using a diffeomorphic normalization method (e.g., ANTS^58^). The resulting spatial transformations (*Native-to-MNI*) should be applied to the individual scalar maps to spatially align them in MNI space. The final spatial orientation, voxel, and image dimensions should coincide with the orientations and dimensions of the reference template employed to build the atlas (ICBM 2009c Nonlinear Asymmetric 1 × 1 × 1 mm^44^). The tools to perform these operations using *antsRegistration* and *antsApplyTransforms* are freely available at the following github repository: https://github.com/connectomicslab/probconnatlas.

Once the scalar maps are transformed to MNI space, the multi-scale probabilistic atlas can be used to perform different operations. The main usage of the atlas is to compute mean, median and standard deviation values of the scalar maps along each WM bundle. This computation outputs connectivity matrices for a selected scale, with connection strengths being the mean, median and standard deviation values of the scalar maps along each scale-specific bundle.

Another possible usage of the atlas is to extract and/or save some specific bundles. The desired bundles should be supplied through a Nifti-1 image or a Comma-Separated Value (.*csv*) text file. If the Nifti-1 image is a binary mask, the bundles intercepting the non-zero values of the mask will be extracted. If the image contains different regions of interest (*ROIs*), only the bundles connecting two or more ROIs will be extracted. In addition, all the bundles intercepting any of the Nifti-1 ROIs can be saved in a single binary mask, which can then be used to restrict voxel-based analyses to bundles connected to certain region of interest (e.g., to WM bundles reaching lesions or tumor masks). In both cases text (csv of Nifti-1 file), if a scalar map is supplied, a table with the mean, median and standard deviation values along the select bundles are stored as well.

## Supplementary information

Supplementary material

## Data Availability

The custom code used to apply the atlas to new subjects is implemented in Python 3.8 and is available at the github repository https://github.com/connectomicslab/probconnatlas. This code needs the multi-scale probabilistic atlas files stored on the Zenodo repository^65^. The used and the current version of the software is 1.0. All the parameters employed to process the datasets are provided in the atlas files.
