# Supplementary material for: A multi-scale probabilistic atlas of the human connectome
Source: Sci Data. 2022 Aug 23;9:516. doi: 10.1038/s41597-022-01624-8 (PMC9399115; doi:10.1038/s41597-022-01624-8)
Supplement: Supplementary file 1 — Supplementary material [file 41597_2022_1624_MOESM1_ESM.pdf]

Supplementary Material:
A multi-scale probabilistic atlas of the human connectome

Yasser Alemán-Gómez[a,b,*], Alessandra Griffa[c,d,e,*], Jean-Christophe Houde[f], Elena Najdenovska[g,h,i], Stefano Magon[j], Meritxell Bach Cuadra[h,g,i], Maxime Descoteaux[f†], Patric Hagmann[a†]

Table of contents:

Supp1. *Whole-brain tractogram filtering* 2

Supp2. *Orientation distribution functions estimation and fiber tracking: Used parameters for the evaluation* 3

Supp3. *Consistency matrix and mean number of streamlines* 5

Supp4. *Cortical coverage* 6

Supp5. *Voxel-wise probability and subject consistency* 9

Supp6. *Reproducibility among acquisitions* 11

Supp7. *Mean FA differences* 14

Supp8. *Mean FA correlations* 17

Supp1. Whole-brain tractogram filtering

The individual whole-brain tractogram for a subject **s** was filtered to extract brain connections $C^{s}_{k,i,j}$. For each pair of GM regions ***(i ,j)*** at parcellation scale ***k***, streamlines that reach both regions were assigned to connection $C^{s}_{k,i,j}$. Since tractography can generate streamlines reaching multiple cortical and subcortical regions, rules were created to determine the connection associated to each scenario. The basic case is a streamline starting from a cortical region *i* and terminating in another cortical region *j*. This streamline is assigned to connection $C^{s}_{k,i,j}$. If, at one endpoint, the streamline goes through more than one consecutive cortical regions without crossing the WM, the last region traversed before termination is taken as the endpoint region. When a streamline goes through a subcortical grey matter region ***g***, such as the globus pallidum or the putamen, the streamline is cut, and a segment is considered to be connection $C^{s}_{k,i,g}$ while the other is connection $C^{s}_{k,g,j}$. If more than one such regions is traversed before reaching the cortex, the algorithm subdivides the streamline in as many segments as required. The basis for this decision is that neurons normally form synapses within the grey matter and, as such, a streamline going through a subcortical region is probably an artifact of the reconstruction of two consecutive WM bundles. Only one exception exists to this rule, which is the case of the thalamic nuclei. When a streamline with an endpoint in a cortical regions *i* crosses multiple thalamic nuclei *t*, *j*, the segments *i-j* and *i-t* form two cortico-thalamic connections $C^{s}_{k,i,j}$ and $C^{s}_{k,i,t}$, while no connection is considered between the thalamic nuclei t and *j*. The basis for this choice is that, to the best of our knowledge, there is no evidence for inter-nuclear connections within the thalamus other than those involving the thalamic reticular nucleus (TRN) [1], whose *in vivo* segmentation is not possible with current methods [2].

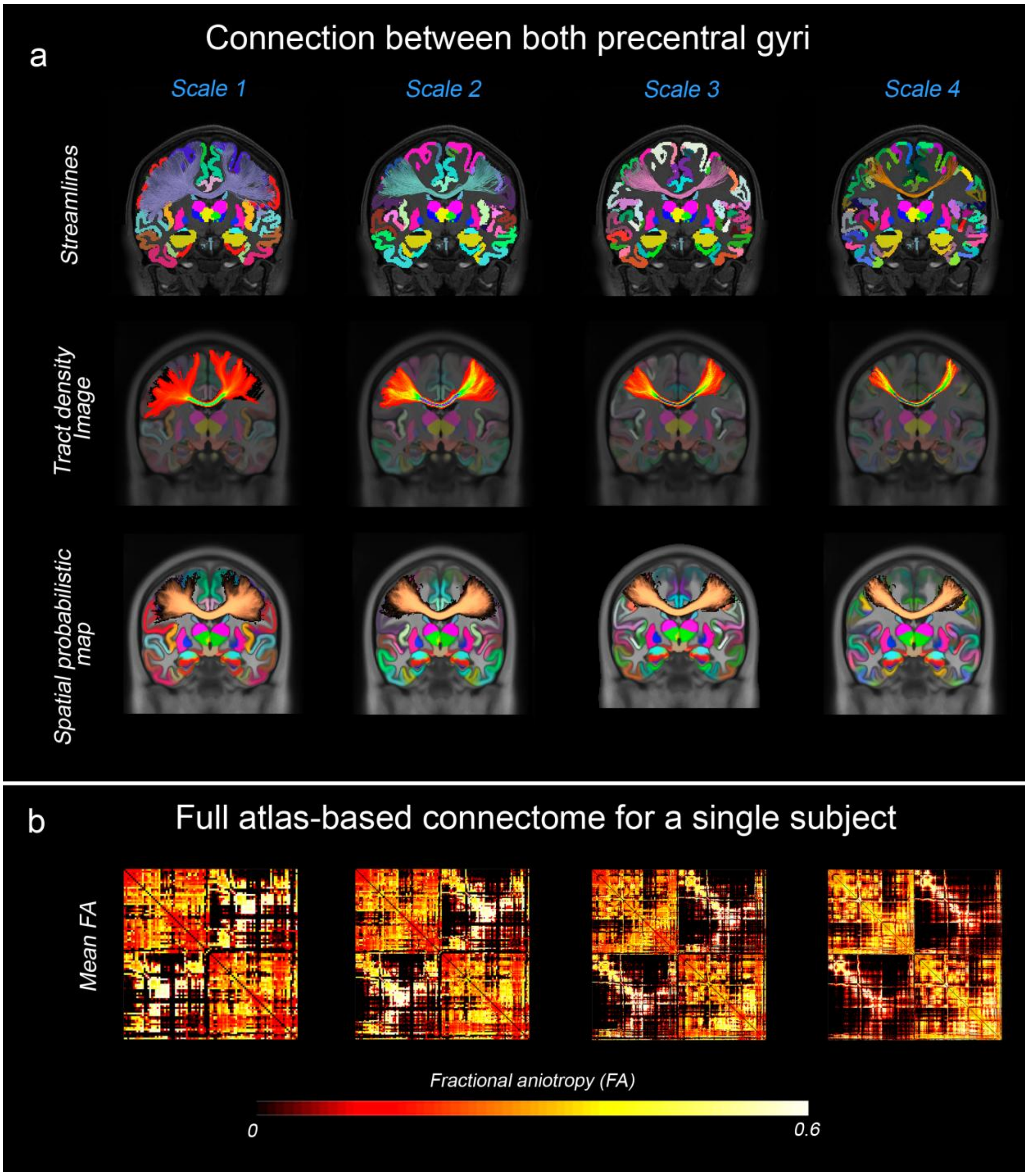

Figure. S1: ***(a)*** *Streamlines, tract density image and probabilistic bundle between both precentral gyri.* ***(b)*** *Mean fractional anisotropy matrices computed for each of the scales included in the atlas.*

Supp2. Orientation distribution functions estimation and fiber tracking: Used parameters for the evaluation

The corrected diffusion weighted image for each subject was employed to estimate the intravoxel fiber orientation distribution function (*fODF*) by using the Constrained Spherical Deconvolution (*CSD*) [3] approach implemented in MRtrix3 (https://www.mrtrix.org/). This technique, based on high-angular resolution diffusion imaging (HARDI) MR data, estimates the orientation of multiple intravoxel fiber populations within regions of complex white matter architecture. The degree for the

decomposition was set to 8 resulting in 45 spherical harmonic functions . This fODFs were used by the *SDSTREAM* (Streamlines by using Spherical Deconvolution) deterministic fiber tracking algorithm to obtain the streamlines distribution for each subject. An *fODF* threshold of 0.1, a maximum angle deviation of 45 degrees, and a step size of 0.5 mm was used. The minimum length of the fiber was set to 300 mm.

These parameters can be verified in the command launched to perform the fiber tractography.

tckgen -force -act *<act5ttFilename>* **-mask** *<dwi_B0_distc_mask>* **-algorithm** *<sd_stream or ifod2>* \
-crop_at_gmwmi *<dwi_fodf>* *<ouput.tck>* **-seed_image** *<wm_mask>* **-select** *4000000* \
-angle *45* **-step** *0.5* **-maxlength** *300* **-trials** *30* **-minlength** *5* **-cutoff** *1*

Finally, the multi-scale structural connectivity matrices, were computed. The connection strength between each pair of regions is given by the number of streamlines connecting them. For the FA scalar map, a structural connectivity matrix was obtained where the connection between each pair of regions is the mean value of this map along the tract connecting them.

Supp3. Consistency matrix and mean number of streamlines

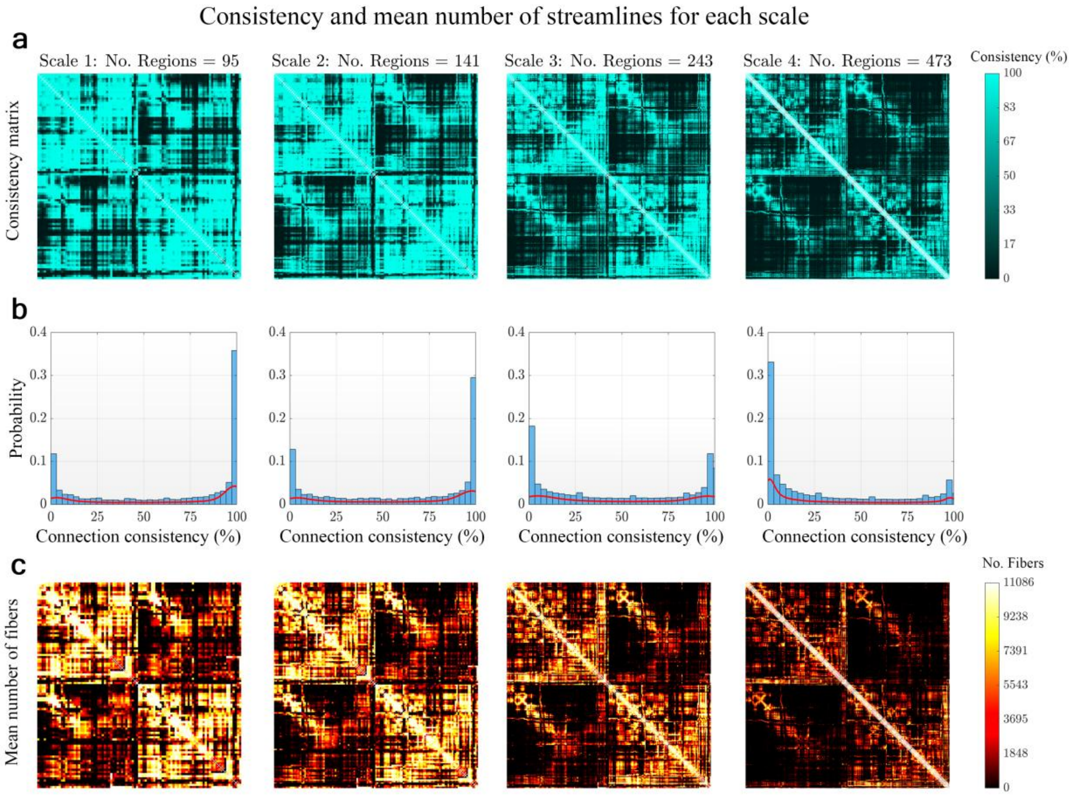

Figure. S2: ***(a)*** *Group-representative connectivity matrices for 4 parcellation scales. Prevalence matrices represent the presence of a connection over the 66 HCP subjects. A connection can be present in 0% to 100% of the subjects.* ***(b)*** *Reproducibility histograms.* ***(c)*** *Connectivity matrices representing the mean number of streamlines for each connection.*

Supp4. Cortical coverage

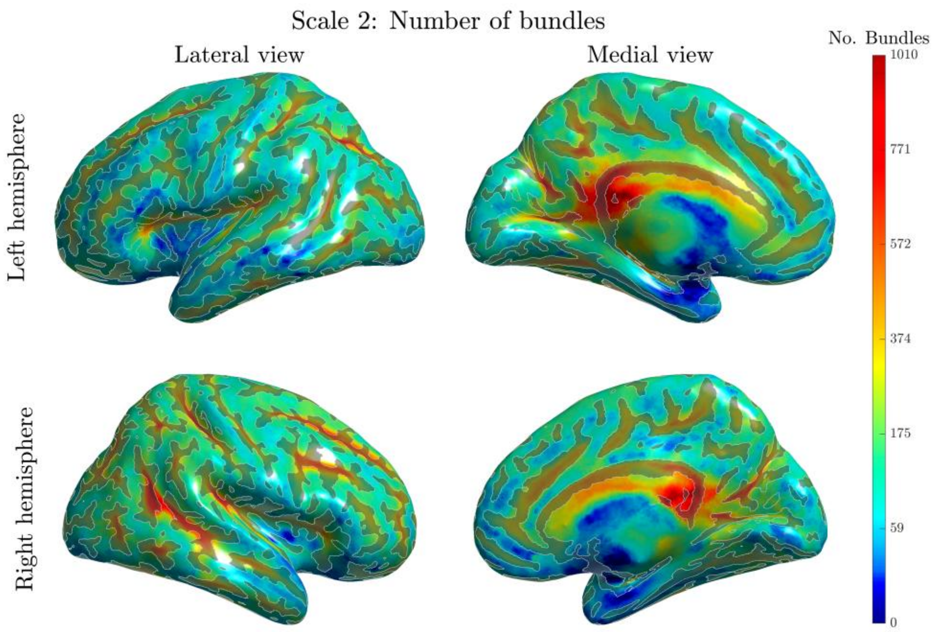

Figure. S3: *Cortical coverage of the WM atlas for scale 2 computed for a single subject of the test-retest dataset. The cortical coverage is represented by the total number of bundles reaching each point of the gray-white matter interface.* **Note:** *The results are displayed over the inflated surface to enhance the visualization in sulcal regions. The cortical regions belonging to sulcal areas are outlined in the figure.*

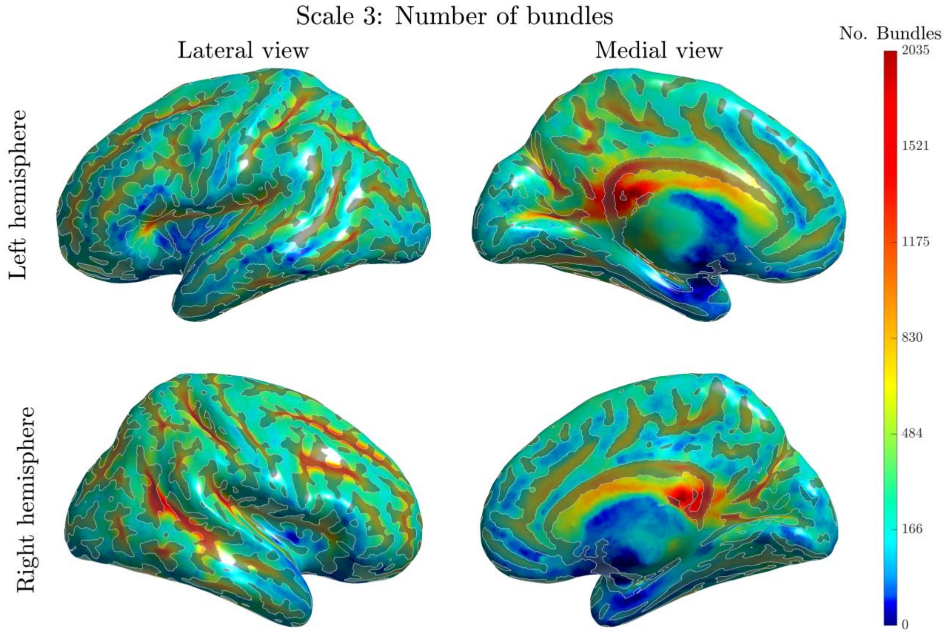

Figure. S4: *Cortical coverage of the WM atlas for scale 3 computed for a single subject of the test-retest dataset. The cortical coverage is represented by the total number of bundles reaching each point of the gray-white matter interface.* **Note:** *The results are displayed over the inflated surface to enhance the visualization in sulcal regions. The cortical regions belonging to sulcal areas are outlined in the figure.*

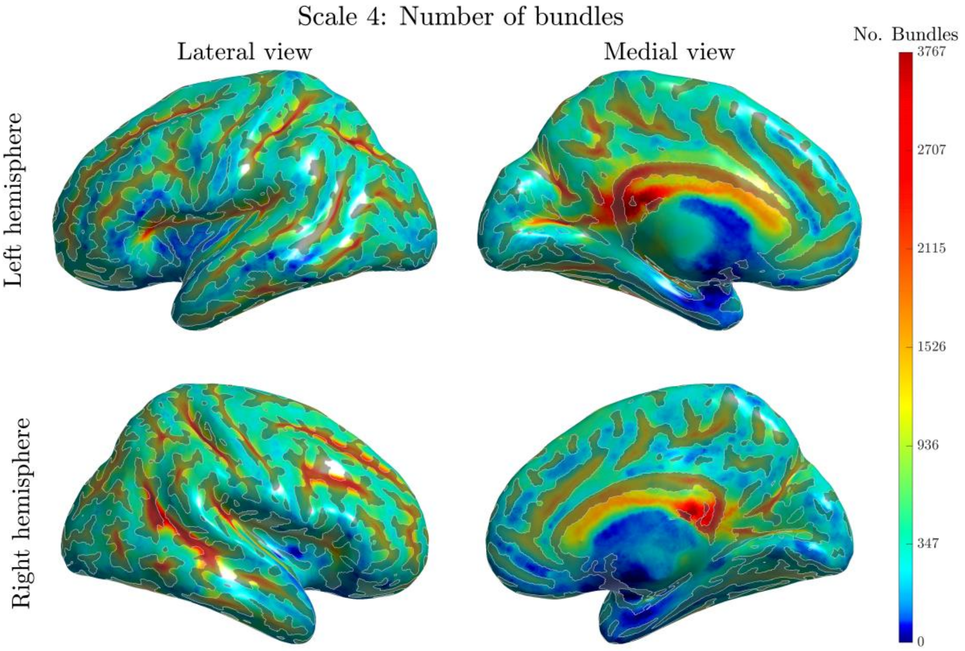

Figure. S5: *Cortical coverage of the WM atlas for scale 4 computed for a single subject of the test-retest dataset. The cortical coverage is represented by the total number of bundles reaching each point of the gray-white matter interface.* **Note:** *The results are displayed over the inflated surface to enhance the visualization in sulcal regions. The cortical regions belonging to sulcal areas are outlined in the figure.*

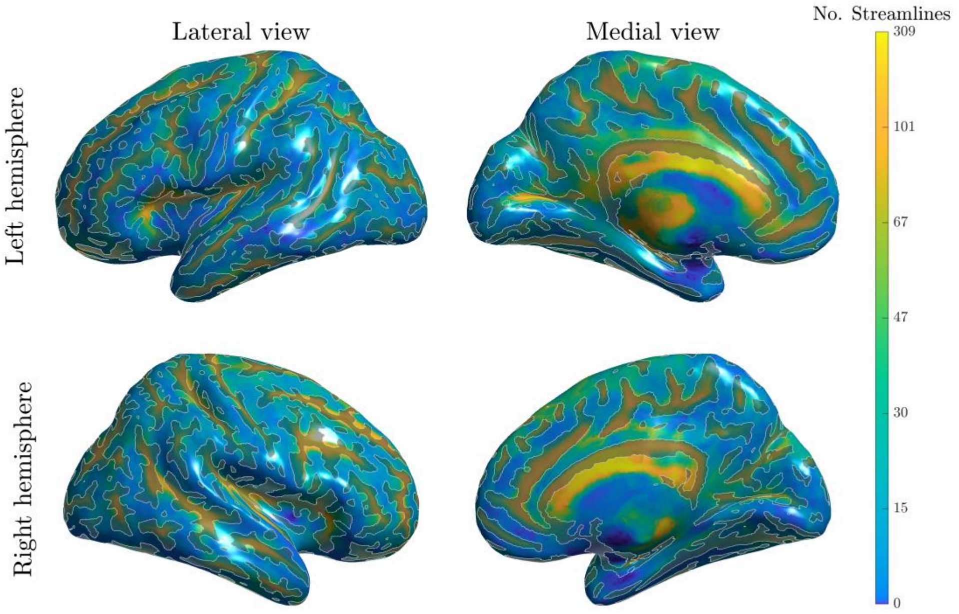

Figure. S6: *Mean number of streamlines, among all the subjects used to build the atlas, reaching each point of the gray-white matter interface.* ***Note:*** *The results are displayed over the inflated surface*

using a logarithmic scale to enhance the visualization in sulcal regions and vertices with low number of streamlines . The cortical regions belonging to sulcal areas are outlined in the figure.

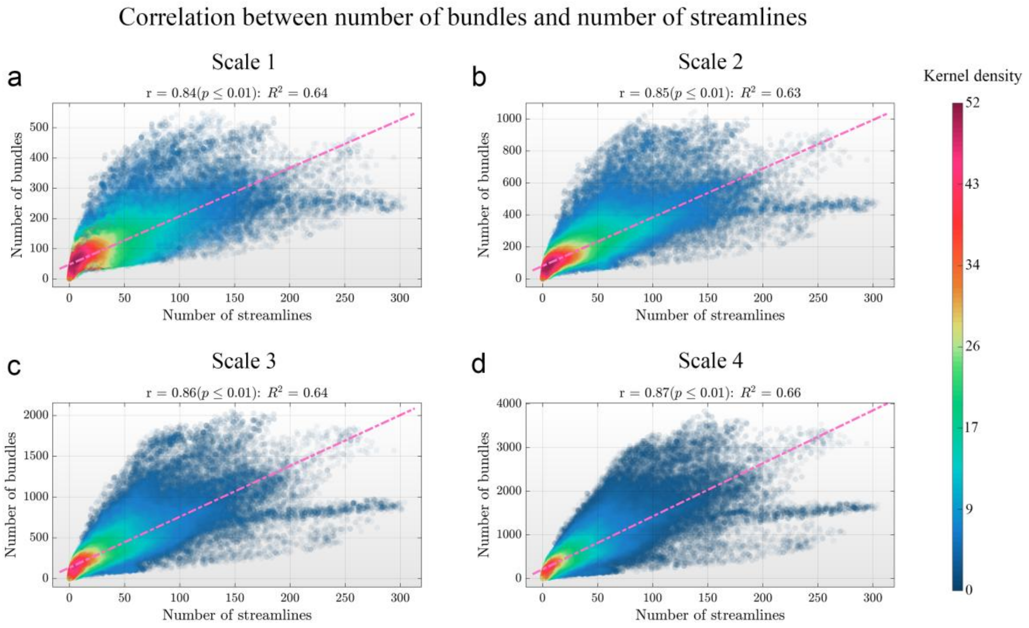

Figure. S7: *Pearson correlation between number of streamlines and the number of bundles on the cortical white surface for each scale.*

Supp5. Voxel-wise probability and subject consistency

In order to find the combination of the two main parameters, the voxel-wise probability (voxth) and the subject consistency (subth), that results in the highest correlation between the atlas-based FA and the tracking-based FA matrices a tuning study was performed

Eleven values for each of both thresholds were selected. The subject consistency thresholds were fixed between from 0 to 100 with a step interval of 10 while the voxel-wise probability ranged from 0 to 1 with a step interval of 0.1.

For each pair of thresholds (voxth, subth), the atlas-based FA matrix was computed for each subject of the HCP test-retest dataset. The resulting matrices were then correlated with the individual tracking-based matrices obtained using either probabilistic or deterministic tractography. Finally, the mean correlation value among all the subjects was computed. The summary of the results for all the combinations are shown in the figure S8. For most of the scales, a voxel-wise threshold of 0.3 and a

consistency value above 30 percent is the optimal combination to obtain a high correlation between the atlas-based and the atlas-based FA matrices.

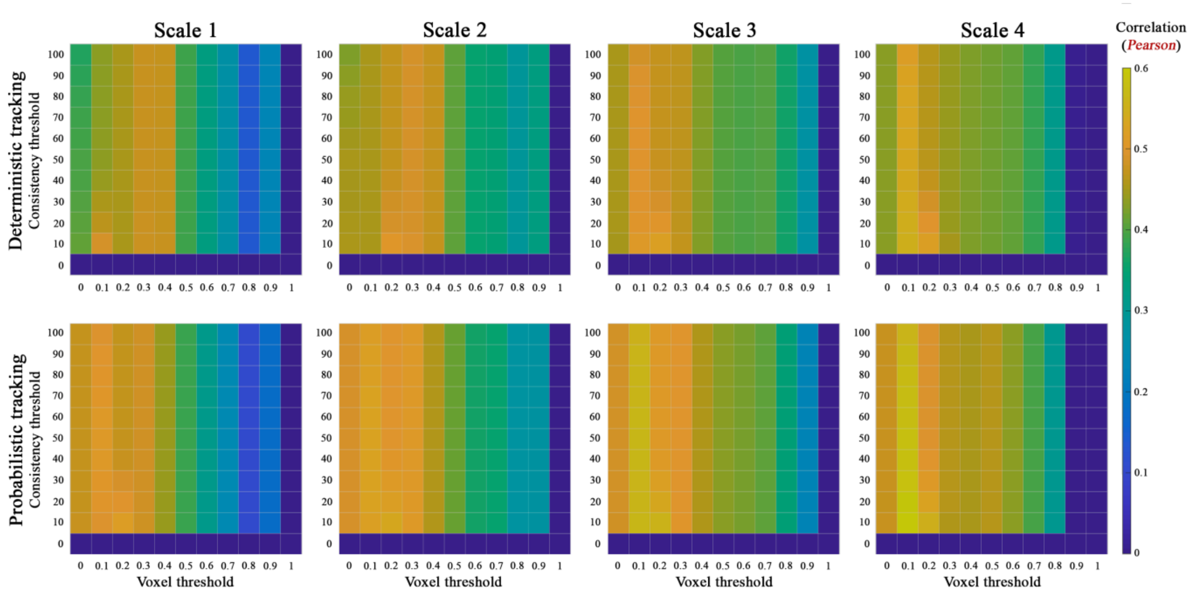

Figure. S8: *Correlation between tracking-based and atlas-based connectivity matrices for each combination of both voxel-wise probability and consistency.*

Supp6. Reproducibility among acquisitions

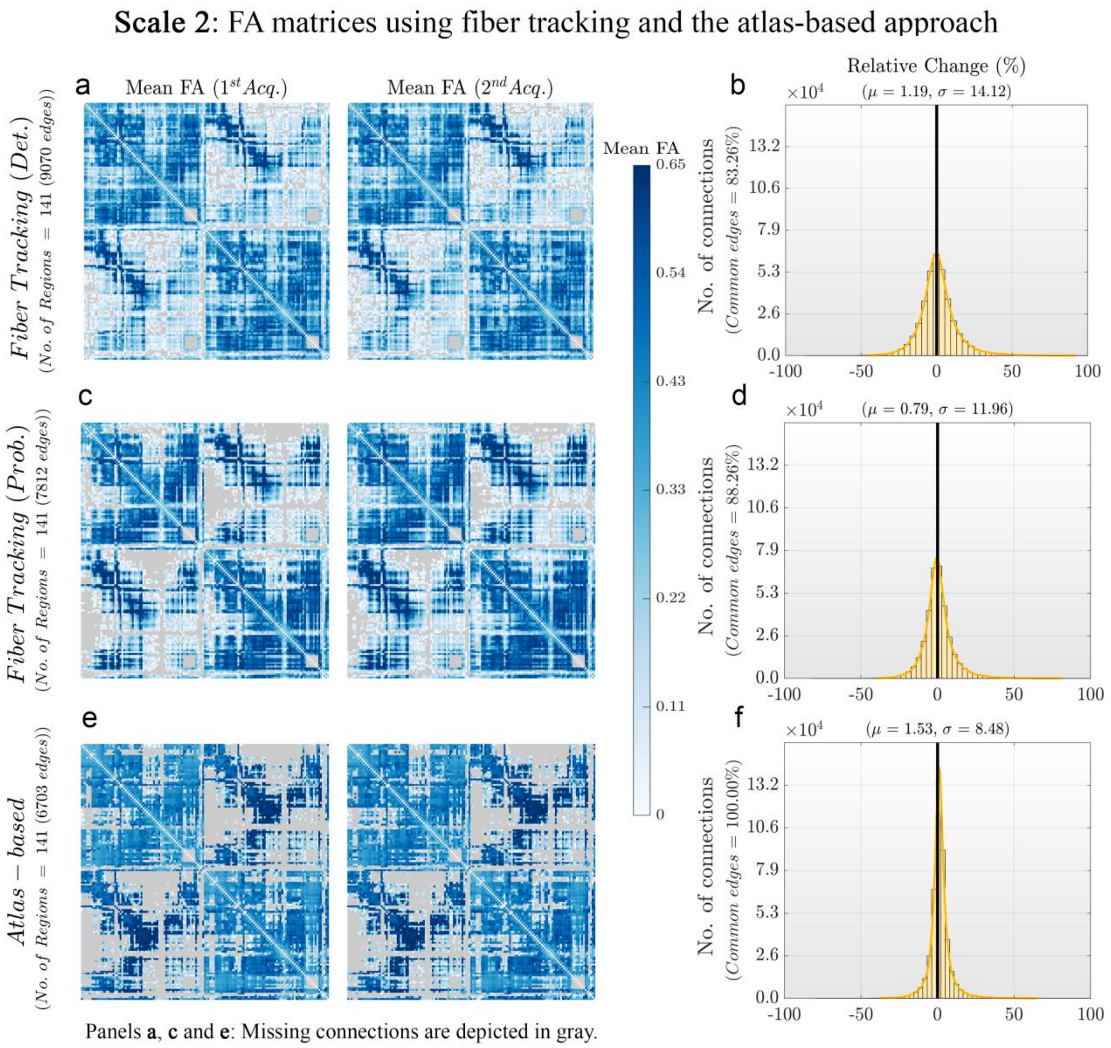

Figure. S9: *Mean FA matrices for both acquisitions and the percentage of difference between them obtained using tracking-based and atlas-based approaches for scale 2.* ***(a)*** *and* ***(c)*** *Mean connectivity matrices computed using two different fiber tracking approaches: 1) deterministic (SD_Stream) and 2) probabilistic (iFOD2).* ***(e)*** *Mean connectivity matrices obtained using the atlas-based approach.* ***Note:*** *These matrices were computed for both acquisition and the connection strength between each pair of regions is given by the mean FA value along the bundle connecting them.* ***(b)***, ***(d)*** *and* ***(f)*** *Histogram of the percentage of difference between the connectivity matrices computed for both acquisitions.*

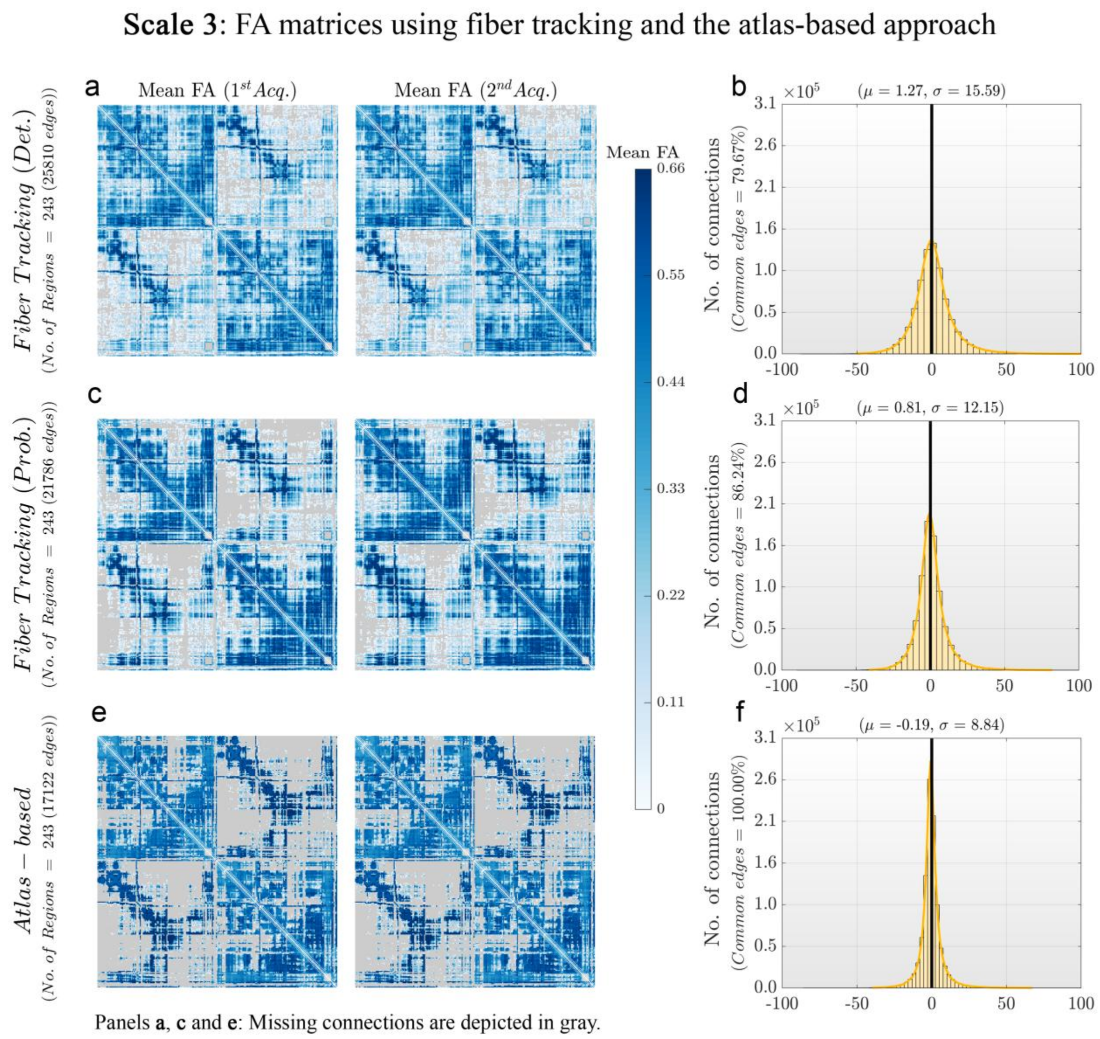

Figure. S10: *Mean FA matrices for both acquisitions and the percentage of difference between them obtained using tracking-based and atlas-based approaches for scale 3.* ***(a)*** *and* ***(c)*** *Mean connectivity matrices computed using two different fiber tracking approaches: 1) deterministic (SD_Stream) and 2) probabilistic (iFOD2).* ***(e)*** *Mean connectivity matrices obtained using the atlas-based approach. Note: These matrices were computed for both acquisition and the connection strength between each pair of regions is given by the mean FA value along the bundle connecting them.* ***(b)***, ***(d)*** *and* ***(f)*** *Histogram of the percentage of difference between the connectivity matrices computed for both acquisitions.*

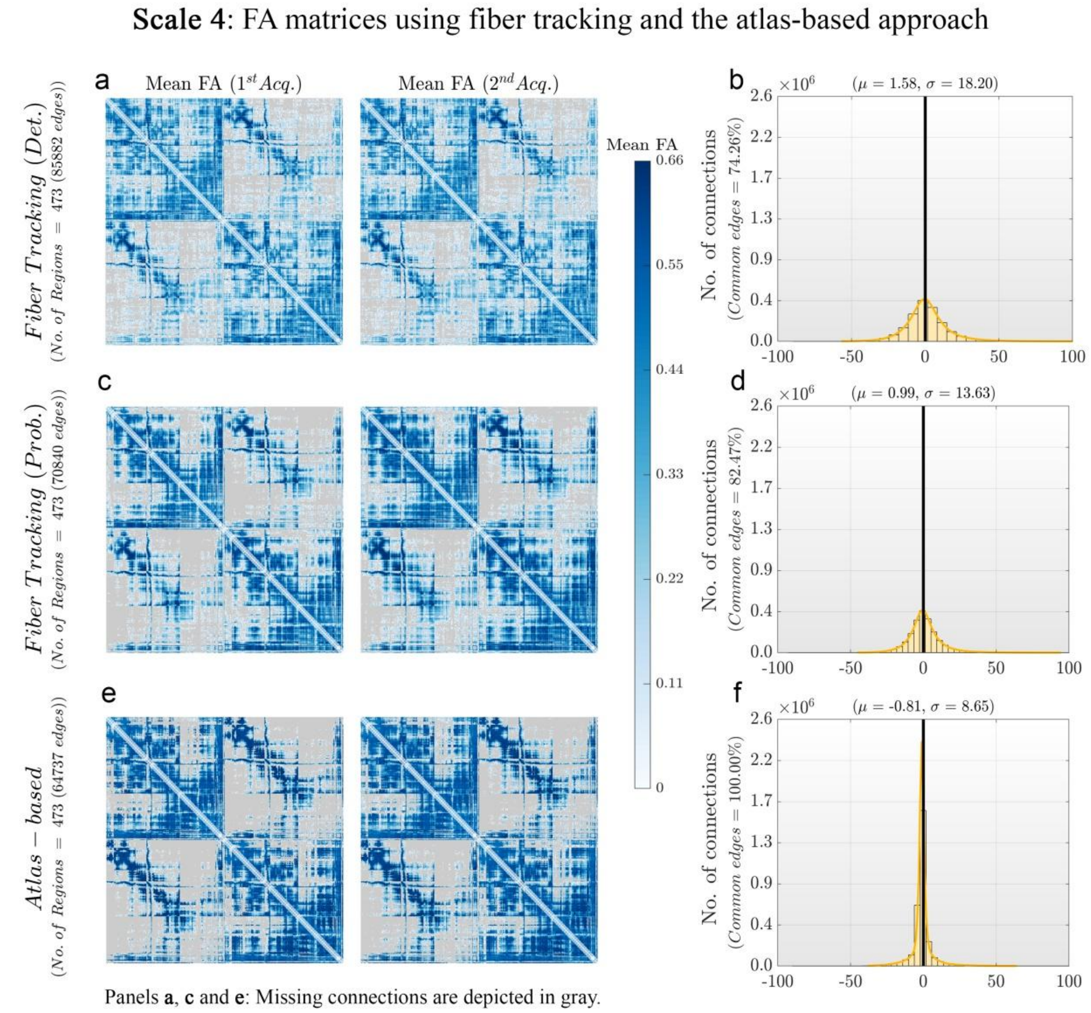

Figure. S11: *Mean FA matrices for both acquisitions and the percentage of difference between them obtained using tracking-based and atlas-based approaches for scale 4.* ***(a)*** *and* ***(c)*** *Mean connectivity matrices computed using two different fiber tracking approaches: 1) deterministic (SD_Stream) and 2) probabilistic (iFOD2).* ***(e)*** *Mean connectivity matrices obtained using the atlas-based approach. Note: These matrices were computed for both acquisition and the connection strength between each pair of regions is given by the mean FA value along the bundle connecting them.* ***(b)***, ***(d)*** *and* ***(f)*** *Histogram of the percentage of difference between the connectivity matrices computed for both acquisitions.*

Supp7. Mean FA differences

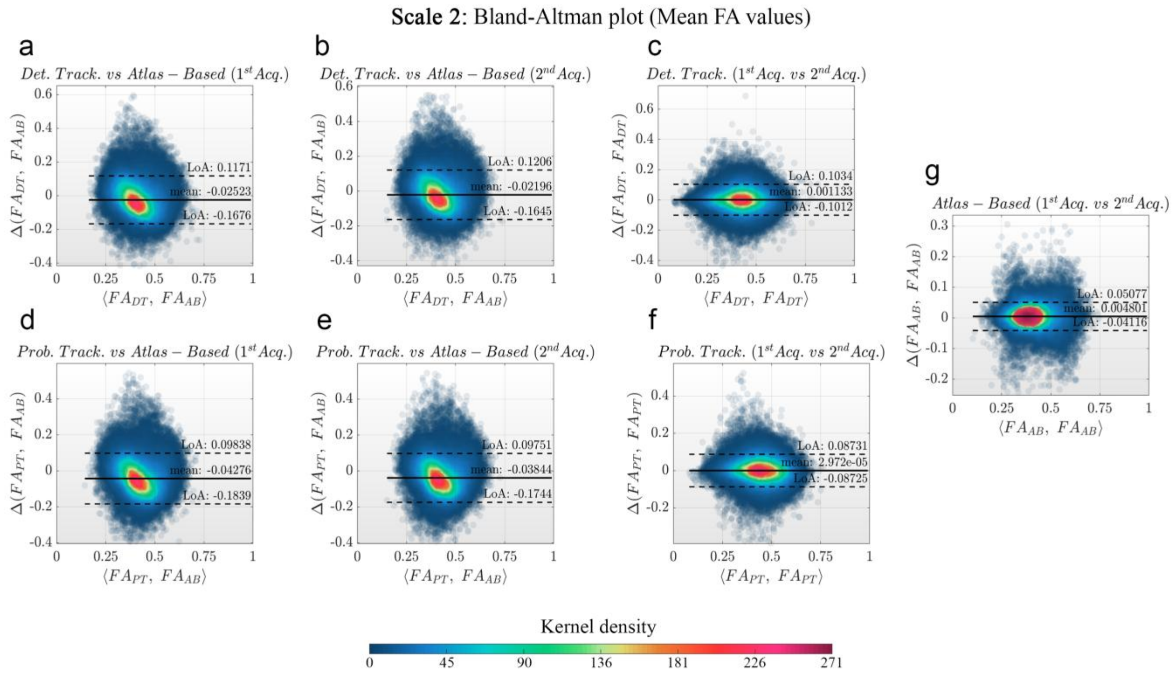

Figure. S12: *Bland-Altman plots displaying the bundles-wise FA differences between tracking-based and atlas-based approaches for the second scale of the developed multi-scale atlas.* ***(a)*** *and* ***(b)*** *Differences between deterministic tracking and atlas-based for both acquisitions of the test-retest dataset.* ***(c)*** *Differences in FA values between both acquisitions when using deterministic fiber tracking.* ***(d)*** *and* ***(e)*** *Differences between probabilistic tracking and atlas-based for both acquisitions of the test-retest dataset.* ***(f)*** *Difference in FA values between both acquisitions when using probabilistic fiber tracking.* ***(g)*** *FA difference between both acquisitions when using the atlas-based approach.* ***Notes:*** *Dashed line represents correlation equal to one. LoA stands for level of agreements. Colors represent the probability density of the sample estimated using the closest 900 points.*

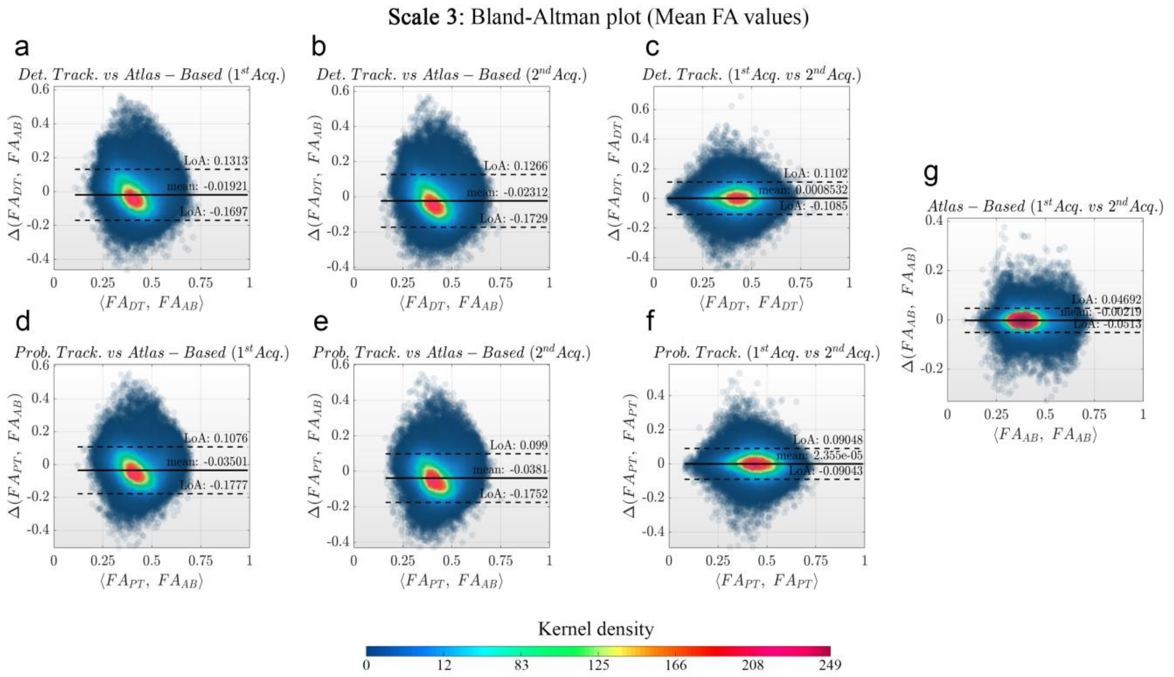

Figure. S13: *Bland-Altman plots displaying the bundles-wise FA differences between tracking-based and atlas-based approaches for the third scale of the developed multi-scale atlas.* ***(a)*** *and* ***(b)*** *Differences between deterministic tracking and atlas-based for both acquisitions of the test-retest dataset.* ***(c)*** *Differences in FA values between both acquisitions when using deterministic fiber tracking.* ***(d)*** *and* ***(e)*** *Differences between probabilistic tracking and atlas-based for both acquisitions of the test-retest dataset.* ***(f)*** *Difference in FA values between both acquisitions when using probabilistic fiber tracking.* ***(g)*** *FA difference between both acquisitions when using the atlas-based approach.* ***Notes:*** *LoA stands for level of agreements. Colors represent the probability density of the sample estimated using the closest 900 points.*

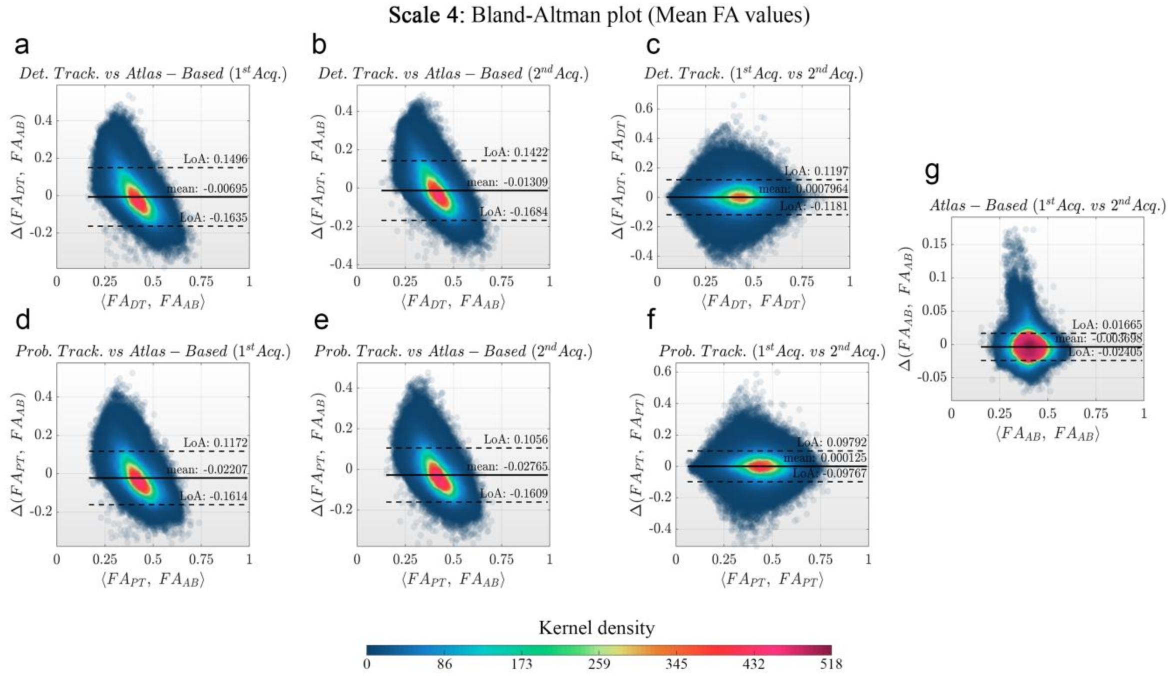

Figure. S14: *Bland-Altman plots displaying the bundles-wise FA differences between tracking-based and atlas-based approaches for the fourth scale of the developed multi-scale atlas.* ***(a)*** *and* ***(b)*** *Differences between deterministic tracking and atlas-based for both acquisitions of the test-retest dataset.* ***(c)*** *Differences in FA values between both acquisitions when using deterministic fiber tracking.* ***(d)*** *and* ***(e)*** *Differences between probabilistic tracking and atlas-based for both acquisitions of the test-retest dataset.* ***(f)*** *Difference in FA values between both acquisitions when using probabilistic fiber tracking.* ***(g)*** *FA differences between both acquisitions when using the atlas-based approach.* ***Notes:*** *LoA stands for level of agreements. Colors represent the probability density of the sample estimated using the closest 900 points.*

Supp8. Mean FA correlations

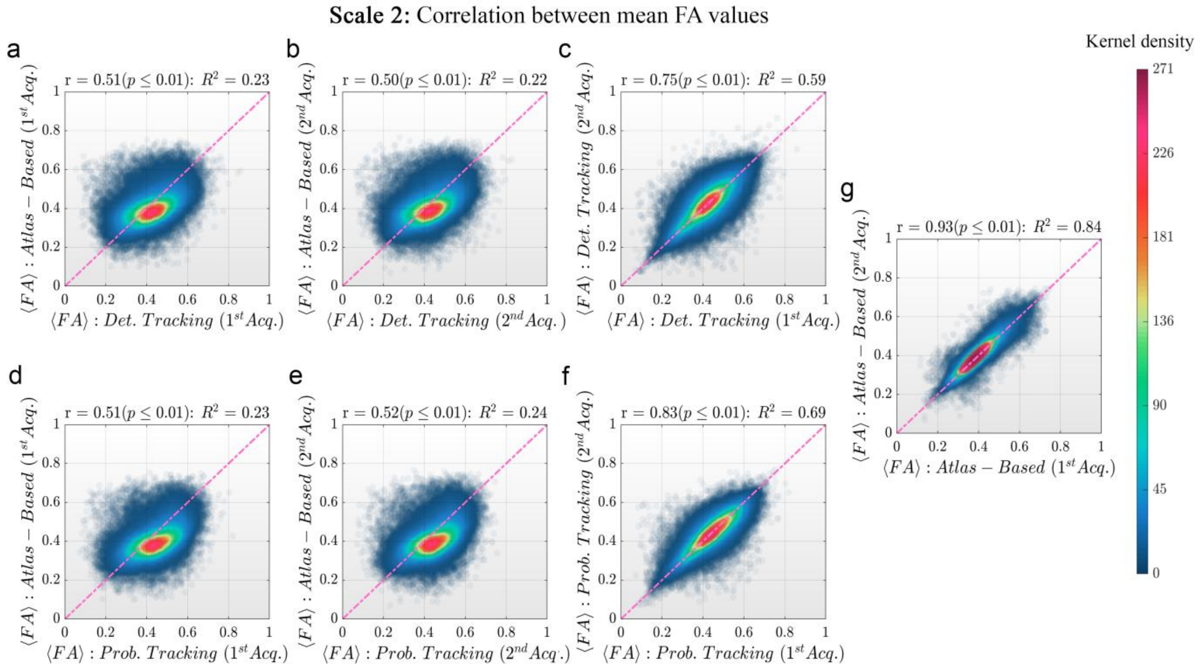

Figure. S15: *Bundles-wise FA correlations between tracking-based and atlas-based approaches for the second scale of the developed multi-scale atlas.* ***(a)*** *and* ***(b)*** *Correlations between deterministic tracking and atlas-based for both acquisitions.* ***(c)*** *Correlation in FA values between both acquisitions using deterministic fiber tracking.* ***(d)*** *and* ***(e)*** *Correlations between probabilistic tracking and atlas-based for both acquisitions.* ***(f)*** *Correlation in FA values between both acquisitions using probabilistic fiber tracking.* ***(g)*** *FA correlation between both acquisitions using the atlas-based approach.* **Notes:** *Dashed line represents correlation equal to one. Colors represent the probability density of the sample estimated using the closest 900 points.*

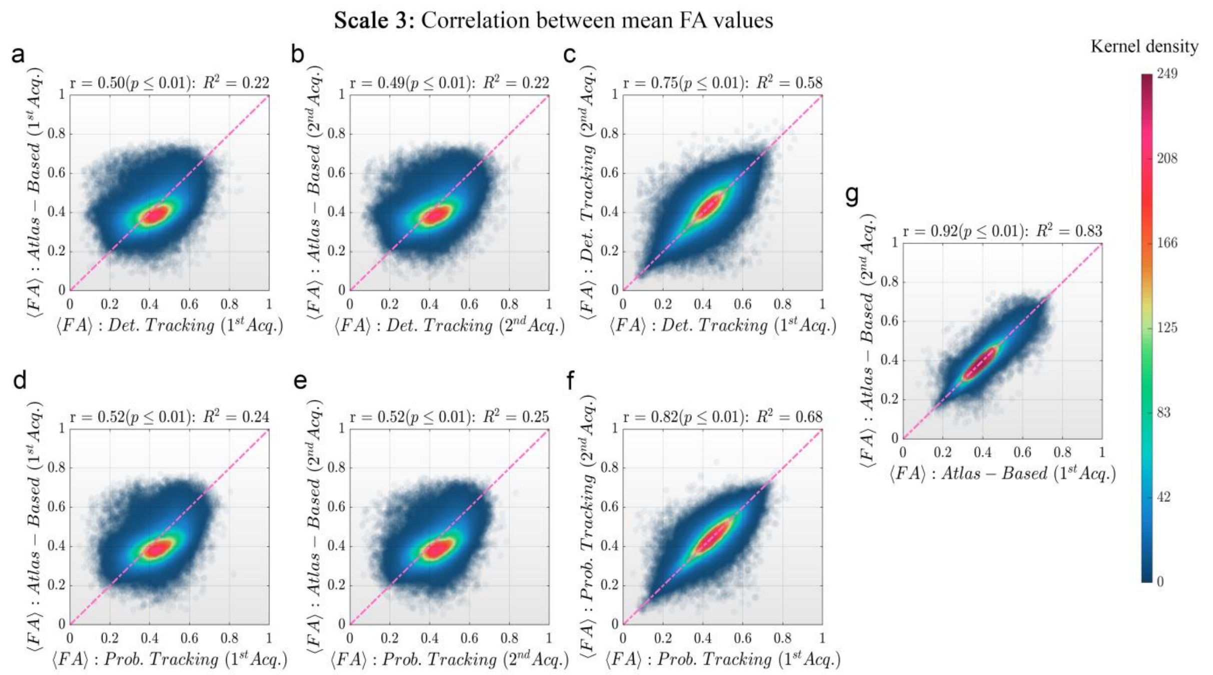

Figure. S16: *Bundles-wise FA correlations between tracking-based and atlas-based approaches for the third scale of the developed multi-scale atlas.* ***(a)*** *and* ***(b)*** *Correlations between deterministic tracking and atlas-based for both acquisitions.* ***(c)*** *Correlation in FA values between both acquisitions using deterministic fiber tracking.* ***(d)*** *and* ***(e)*** *Correlations between probabilistic tracking and atlas-based for both acquisitions.* ***(f)*** *Correlation in FA values between both acquisitions using probabilistic fiber tracking.* ***(g)*** *FA correlation between both acquisitions using the atlas-based approach.* ***Notes:*** *Dashed line represents correlation equal to one. Colors represent the probability density of the sample estimated using the closest 900 points.*

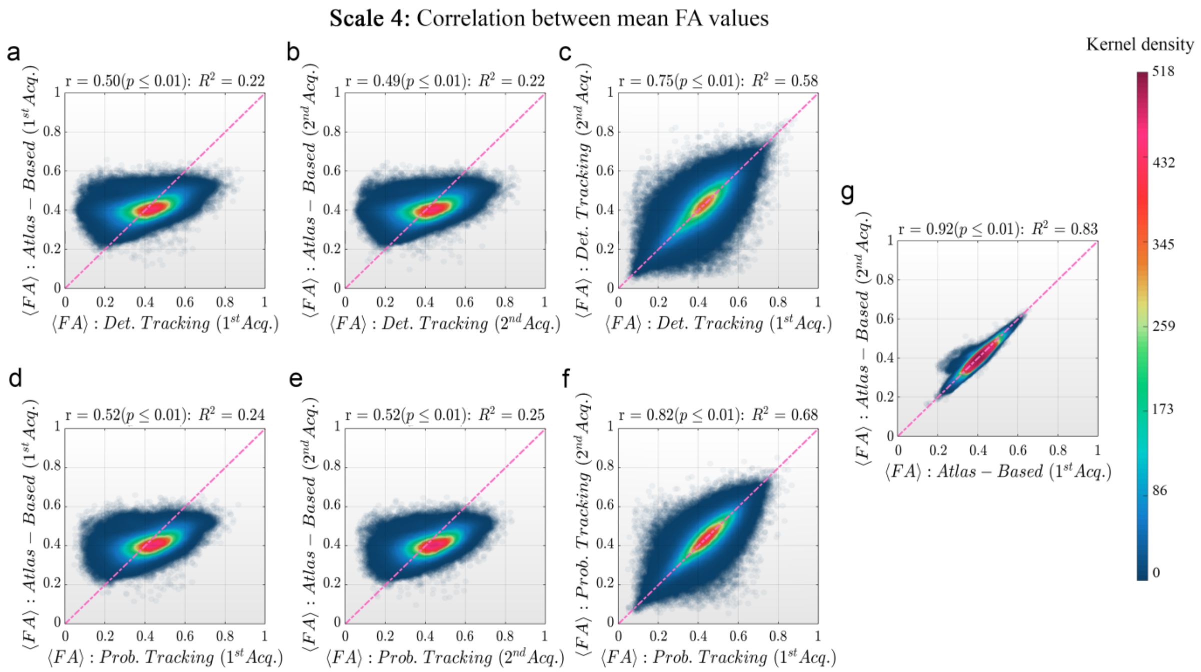

Figure. S17: *Bundles-wise FA correlations between tracking-based and atlas-based approaches for the fourth scale of the developed multi-scale atlas.* ***(a)*** *and* ***(b)*** *Correlations between deterministic tracking and atlas-based for both acquisitions.* ***(c)*** *Correlation in FA values between both acquisitions using deterministic fiber tracking.* ***(d)*** *and* ***(e)*** *Correlations between probabilistic tracking and atlas-based for both acquisitions.* ***(f)*** *Correlation in FA values between both acquisitions using probabilistic fiber tracking.* ***(g)*** *FA correlation between both acquisitions using the atlas-based approach.* ***Notes:*** *Dashed line represents correlation equal to one. Colors represent the probability density of the sample estimated using the closest 900 points.*

References

1 Crabtree, J. W. & Isaac, J. T. New intrathalamic pathways allowing modality-related and cross-modality switching in the dorsal thalamus. *The Journal of neuroscience : the official journal of the Society for Neuroscience* **22**, 8754-8761 (2002).

2 Battistella, G. *et al.* Robust thalamic nuclei segmentation method based on local diffusion magnetic resonance properties. *Brain structure & function* **222**, 2203-2216, doi:10.1007/s00429-016-1336-4 (2017).

3 Tournier, J. D., Calamante, F. & Connelly, A. Robust determination of the fibre orientation distribution in diffusion MRI: non-negativity constrained super-resolved spherical deconvolution. *NeuroImage* **35**, 1459-1472, doi:10.1016/j.neuroimage.2007.02.016 (2007).
